# An *Auto-RS* Signature for Prognostic Stratification and Drug Sensitivity Prediction in Osteosarcoma

**DOI:** 10.3390/genes17070737

**Published:** 2026-06-26

**Authors:** Qingzhu Liu, Ke Xu, Cong Zhou, Qikui Zhu, Junqin Lu, Yuqiao Tang, Chun Zhang, Wukun Xie, Guojiu Fang, Dasheng Tian, Juehua Jing, Yize Li, Wenxiu Duan, Hongsheng Wang, Yihui Bi

**Affiliations:** 1 Department of Orthopaedics, The Second Affiliated Hospital of Anhui Medical University, Hefei 230601, China; 2445011963@stu.ahmu.edu.cn (Q.L.); xuke9723@163.com (K.X.); 18870425648@163.com (C.Z.); xinlan163668@163.com (C.Z.); drwukunxie@163.com (W.X.); tiandasheng@ahmu.edu.cn (D.T.); jjhhu@sina.com (J.J.); wxduan9@ustc.edu.cn (W.D.); 2 Institute of Orthopaedics, Research Center for Translational Medicine, The Second Affiliated Hospital of Anhui Medical University, Hefei 230601, China; 3 State Key Laboratory of Oral & Maxillofacial Reconstruction and Regeneration, School & Hospital of Stomatology, Wuhan University, Wuhan 430079, China; qikuizhu@163.com; 4 Department of Orthopedics, The First People’s Hospital of Lianyungang City, Lianyungang 222061, China; junqinlu1989@163.com; 5 College of Life Sciences, Wuhan University, Wuhan 430072, China; 2021302042013@whu.edu.cn; 6 Department of General Surgery, Shanghai Fengxian Central Hospital, Shanghai 201400, China; fgj1202@163.com; 7 Department of Medicine, Washington University in St. Louis, St. Louis, MO 63130, USA; yize.li@wustl.edu; 8 Department of Traditional Chinese Medicine, Songjiang Research Institute, Shanghai Key Laboratory of Emotions and Affective Disorders, Songjiang Hospital Affiliated to Shanghai Jiao Tong University School of Medicine, Shanghai 201600, China; 9 Department of Neurosciences, School of Medicine, Case Western Reserve University, Cleveland, OH 44106, USA

**Keywords:** osteosarcoma, prognostic biomarker, risk stratification, machine learning, single-cell analysis, drug sensitivity prediction

## Abstract

Background: Metastasis and poor chemotherapy response have stagnated therapeutic progress in osteosarcoma (OS) for the past three decades. Defining the transition from localized to metastatic OS before overt dissemination is fundamental for improving survival. However, effective early diagnostic tools remain scarce, largely due to limited exploitation of the metastasis-associated tumor microenvironment’s own record of prior environmental and stress exposures encoded in cell-intrinsic transcriptional states. Here, we employed a supervised machine learning framework with iterative resampling and multi-stage model selection to identify molecular markers associated with metastasis in osteosarcoma and to develop a computational signature, *Auto-RS*. Methods: Transcriptomic and clinical data from 139 OS patients with ≥5 years of follow-up were analyzed. A LASSO–Cox framework was applied to derive a gene expression-based risk score, *Auto-RS*, from which a nomogram integrating age and sex was generated for individualized prognosis. Model interpretability was assessed across six independent single-cell OS patient datasets, and drug sensitivity predictions were inferred by integrating *Auto-RS* with the Precily algorithm to uncover actionable therapeutic vulnerabilities. Results: *Auto-RS*, constructed from the expression of four autophagy genes (*BNIP3*, *MYC*, *PEA15*, and *SAR1A*), served as an independent prognostic factor for overall survival (HR = 1.091; 95% CI, 1.047–1.136; *p* < 0.001). Time-dependent ROC analysis showed that *Auto-RS* was the most accurate single predictor (AUC = 0.88), exceeding metastasis (0.83), sex (0.45), and age (0.39). A basic prognostic model (BpM) incorporating metastasis status yielded a C-index of 0.741 (95% CI, 0.679–0.803). The addition of *Auto-RS* (CpM) improved discrimination (C-index = 0.788; 95% CI, 0.731–0.845), whereas a model without metastasis information (ApM) retained predictive ability (C-index = 0.709; 95% CI, 0.640–0.778). Single-cell analysis confirmed that *Auto-RS* features aligned with known metastatic trajectories, reflecting the transition from proliferative to invasive tumor states and highlighting coordinated programs among cancer-associated fibroblasts and immune cells. Drug sensitivity integration through *Precily* identified gemcitabine and cytarabine as FDA-approved agents predicted in silico to show greater sensitivity in the high-risk subgroup. Conclusions: We identified autophagy-mediated transcriptional ‘stress fingerprints’ that are tightly associated with OS metastasis. The *Auto-RS* signature, composed of *BNIP3*, *MYC*, *PEA15*, and *SAR1A*, enables early therapeutic stratification of patients independent of overt metastatic status. Moreover, *Auto-RS* delineates key molecular underpinnings of OS metastasis at single-cell resolution. As a practical laboratory tool, *Auto-RS* may represent a step toward improved risk stratification, where advances in metastasis prediction and therapeutic guidance converge to improve outcomes in OS.

## 1. Introduction

Osteosarcoma (OS) is rare but remains the most common primary malignant bone tumor, with an annual incidence of 0.2–3 cases per 100,000 children and 0.8–11 cases per 100,000 adolescents, which coincides with periods of rapid skeletal growth [1,2]. In the United States, an estimated 800–900 new cases are diagnosed annually, mostly in children and young adults. Current guidelines recommend multi-agent chemotherapy combined with complete surgical resection, which has increased 5-year survival for localized disease to ~60% [3]. However, despite decades of therapeutic exploration, survival for patients with metastatic or recurrent OS has stagnated at <20% [4].

Most OS patients harbor clinically undetectable micro-metastases at presentation, a hallmark attributed to the tumor’s aggressive biology and profoundly unstable genomic architecture [5,6,7,8]. Recent consensus suggests that transformative advances in treatment are unlikely to result from the intensification of antineoplastic chemotherapy, as the rarity of OS worldwide precludes large-scale trials of newer drugs, and companies prefer to avoid direct testing in children [9,10]. Instead, the post-genomic era is driving the development of stratification systems that categorize patients based on prognostic or biological features of OS, with the potential to impact outcomes [1,2,10,11]. Yet, existing prognostic models for OS metastasis remain limited and suboptimal because they lack systematic screening and in-depth interpretability [12].

One promising biological axis for such an interpretable, mechanism-anchored stratification is autophagy. Briefly, autophagy acts as a context-dependent, largely cytoprotective stress response pathway in OS [13,14]: it supports tumor cell survival under hypoxia, nutrient deprivation and chemotherapeutic stress [13,15]; contributes to resistance against doxorubicin, cisplatin and methotrexate [16]; and has been linked to invasion and metastatic competence [17], while in certain contexts it can also restrain malignant progression [14]. This dual, stress-adaptive behavior, and its tight coupling to the metastatic and treatment-resistant phenotypes that dominate OS outcomes, motivated our focus on autophagy genes as candidate readouts of metastatic biology.

Building on this rationale, we sought to establish an effective tool for early prediction of OS prognosis and drug sensitivity, and to functionally map its biological relevance at the single-cell level within the tumor microenvironment.

## 2. Results

### 2.1. OS Patient Cohorts

We utilized ongoing Therapeutically Applicable Research To Generate Effective Treatments (TARGET) study information, curated by the U.S. National Cancer Institute (NCI), which comprises the genomic profiles of 88 clinically annotated patients. In parallel, we incorporated whole-genome expression data from 53 high-grade OS cases provided by the EuroBoNet consortium and GEO database (GSE21257) [18]. Two TARGET cases were excluded due to missing survival data. The baseline clinicopathological characteristics of both cohorts are summarized in Table 1.

### 2.2. Autophagy Gene Dysregulation Characterizes Metastatic OS

The patients with metastasis had significantly shorter survival than those without (TARGET, *p* = 0.0018, GSE21257, *p* = 8.4 × 10^−7^; Figure S1A,B). Multivariable Cox regression confirmed metastasis as an independent predictor of poor prognosis (*p* < 0.001), whereas age and sex were not statistically significant (sex, *p* = 0.574; age, *p* = 0.976; Figure S1C). On this basis, we developed a basic prognostic model (BpM) as a nomogram to estimate 1-, 3-, and 5-year overall survival (Figure S1D). Although age and sex did not independently affect outcome, both were retained given prior evidence of clinical relevance in OS, thereby enhancing real-world applicability [2]. Metastatic status carried the greatest prognostic weight, producing the widest spread of total scores, with survival probability declining steeply as scores increased (Table S1). Model calibration using 1000 Bootstrapping showed close agreement between the predicted and observed outcomes at higher risk levels (survival probability 0.0–0.6), with deviations emerging at the opposite end of the risk spectrum (survival probability 0.6–1.0) (Figure S1E).

We next profiled the transcriptional differences between metastatic and non-metastatic cases to identify molecular features that could refine the BpM, particularly in cases lacking explicit metastasis annotation, enabling more individualized risk stratification. Our earlier attempt to derive model genes from global transcriptomic data was abandoned due to limited interpretability, as discussed later (see Section 3). Given the well-established role of the autophagy program in metastatic progression across cancers [19,20,21,22,23,24,25,26], we focused on the dysregulation of autophagy genes in OS. To this end, expression data were integrated, with batch effects corrected using removeBatchEffect (limma) and assessed with BatchServer (Figure S1F,G). Cross-referencing with the Human Autophagy Database yielded 193 autophagy genes (Figure S1H, Tables S2 and S3). Differentially expressed gene (DEG) analysis (limma) identified 12 upregulated and 17 downregulated autophagy genes in metastatic versus non-metastatic cases (Figure S1I). We further visualized the gene-wise expression trajectories stratified by metastasis status, and examined correlations of DEGs with sex and survival status (Fustat) (Figure S1J). These analyses underscored both the sensitivity of autophagy gene expression to metastatic progression and considerable heterogeneity among the patients.

### 2.3. Screening Prognostic Signature-Associated Autophagy Genes

**Figure 1:**
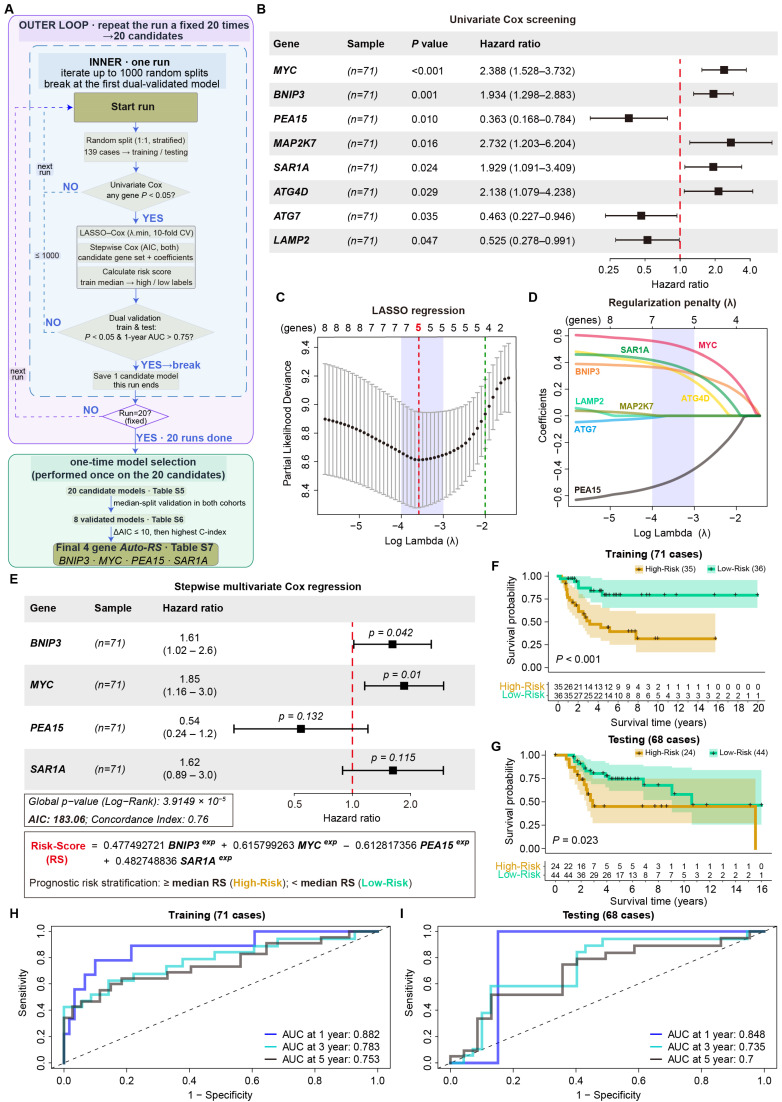
A supervised machine learning framework with iterative resampling and multi-stage model selection identifies prognostic autophagy genes. (**A**) The workflow of the screening framework. The pipeline comprises an inner run that iterates over up to 1000 random training/testing splits and stops (break) at the first dual-validated model; this run is repeated a fixed 20 times to accumulate 20 candidate models (Table S5), followed by a one-time selection step (20 to 8 models by cohort-level validation; final model by ΔAIC ≤ 10 and highest C-index; Tables S6 and S7). Rectangles denote operational steps, diamonds decision nodes, and arrows flow direction (detailed in Section 4). (**B**) A forest plot of eight autophagy genes significantly associated with OS survival by univariate Cox analysis (hazard ratio with 95% CI, Wald test). (**C**) Tenfold cross-validation for the LASSO regression model. The partial likelihood deviance curve is plotted against log(λ); the dotted vertical lines represent the values of λ corresponding to the minimum deviance (red) and the one-standard-error criterion (green). (**D**) The LASSO coefficient profiles of the candidate genes. Each colored line represents one gene; the coefficients shrink toward zero with increasing λ, retaining only the most predictive genes. (**E**) Multivariate Cox regression identifying four independent prognostic genes (*BNIP3*, *MYC*, *PEA15*, *SAR1A*) with corresponding hazard ratios (95% CI, Wald test). The *Auto-RS* was calculated as the sum of gene expression weighted by Cox coefficients, dichotomized by median into high- vs. low-risk groups. The global *p* value was obtained by the log-rank test. (**F**,**G**) The Kaplan–Meier survival curves of the training set (*n* = 71) and testing set (*n* = 68), showing significantly worse survival in the high-risk group (log-rank *p* < 0.05). These internal partitions illustrate model construction; strict external validation (independent-cohort lock-and-transfer and a third cohort) is presented in Supplementary Figure S9C–H. (**H**,**I**) Time-dependent ROC curves assessing model discrimination at 1-, 3-, and 5-year survival. The AUC values demonstrate high predictive accuracy in both the training and testing cohorts.

To derive a parsimonious yet robust prognostic signature from autophagy genes, we developed a supervised machine learning framework with iterative resampling and multi-stage model selection that integrated least absolute shrinkage and selection operator (LASSO) regression with multivariate Cox proportional hazards modeling. This enabled simultaneous dimensionality reduction and capture of survival-associated signals. The workflow proceeds in three stages: (i) random partitioning of the integrated cohorts into training and testing subsets (Figure 1A and Section 4, Table S4); (ii) identification of prognosis-associated autophagy genes and construction of a risk score (*Auto-RS*) formula (Figure 1B–E); and (iii) evaluation of predictive performance in both subsets (Figure 1F–I). During feature selection, LASSO regression was employed to mitigate overfitting and to address the multicollinearity inherent in high-dimensional transcriptomic data (Figure 1C). By imposing a regularization penalty, LASSO shrinks the coefficients of less informative variables toward zero, yielding a sparse set of candidate predictors. The analysis was conducted using the glmnet, with 10-fold cross-validation to determine the optimal regularization parameter (λ). Genes with non-zero coefficients at λ_min (the value minimizing the cross-validated error) were retained (Figure 1D). To further improve interpretability and reduce complexity, LASSO-selected features were subjected to bidirectional stepwise multivariate Cox regression, guided by minimization of the Akaike Information Criterion (AIC) (Figure 1E). This refinement step prioritized the most informative predictors while penalizing excessive model complexity.

This framework screened 20 candidate gene sets (Table S5), and those that can stratify survival (*p* < 0.05) were retained (Table S6). Among these, models with ΔAIC ≤ 10 were further considered, and the one with the highest concordance index (C-index) was ultimately selected (Table S7). Univariate Cox analysis identified eight survival-associated autophagy genes, and LASSO regression reduced these to five candidates (Figure 1B–D). Stepwise multivariate Cox regression further refined the model, resulting in a four-gene signature comprising *BNIP3*, *MYC*, *PEA15*, and *SAR1A* (Figure 1E). Among them, *PEA15* (hazard ratio = 0.54) acted as a protective factor, while *BNIP3* (hazard ratio = 1.61), *MYC* (hazard ratio = 1.85), and *SAR1A* (hazard ratio = 1.62) were risk factors. The *Auto-RS*, calculated by weighting gene expression with Cox coefficients (β), effectively stratified the patients in both the training and testing cohorts (*p* < 0.05; Figure 1F,G), with strong predictive accuracy (training AUCs: 0.882, 0.783, 0.753; testing AUCs: 0.848, 0.735, 0.700; Figure 1H,I). Notably, *Auto-RS* reclassified 19 metastatic patients as low-risk and 33 non-metastatic patients as high-risk, indicating its ability to capture heterogeneity beyond clinical metastasis (Figure S2A,B, Table S8). Kaplan–Meier and time-dependent ROC analyses further confirmed its robust predictive performance in both the integrated cohort (AUCs: 0.869, 0.742, 0.701; Figure S2C,D) and the individual group (Figure S2E–H). To assess generalizability beyond this internal resampling, the locked four-gene signature was further evaluated by strict external validation (Supplementary Figure S9C–H; see Section 3).

In summary, *BNIP3*, *MYC*, *PEA15*, and *SAR1A* constitute a four-gene prognostic signature that refines risk stratification and captures molecular heterogeneity in OS.

### 2.4. Auto-RS Is an Independent Prognostic Factor

**Figure 2:**
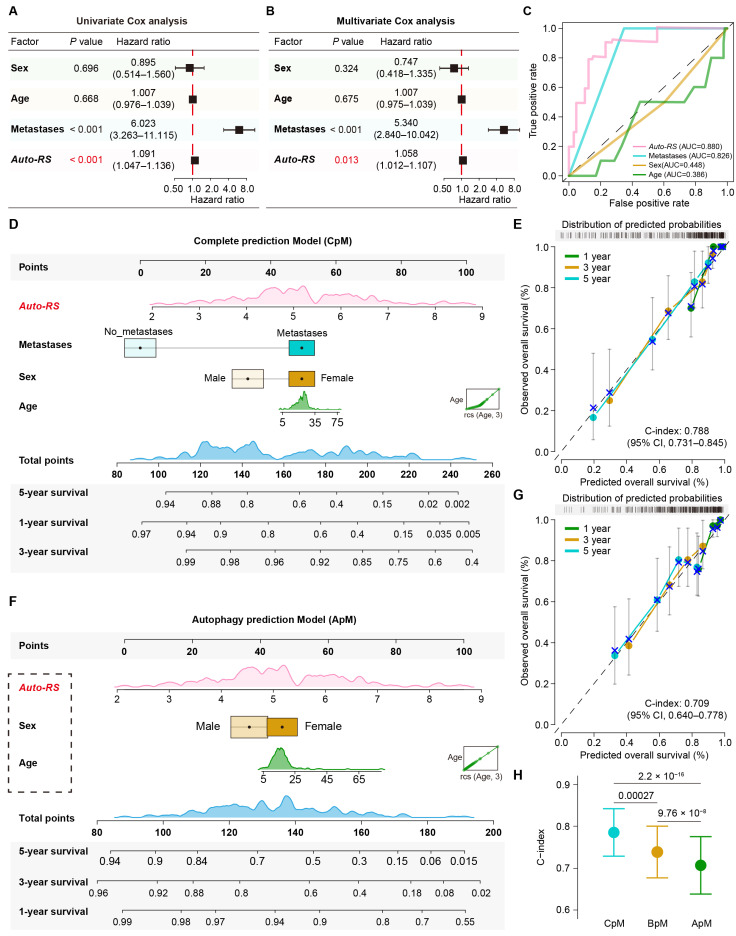
Prognostic models integrating *Auto-RS* and clinical variables in OS. (**A**) Univariate Cox regression of OS survival for sex, age, *Auto-RS*, and metastasis. The horizontal lines indicate the 95% CI of hazard ratios. The *p* values were calculated using a two-sided likelihood ratio test. (**B**) Multivariate Cox regression incorporating sex, age, *Auto-RS*, and metastasis. (**C**) ROC curves comparing predictive performance of *Auto-RS* and clinical variables. AUC indicates discrimination; values closer to 1 reflect higher accuracy. (**D**) A nomogram of the complete prediction model (CpM) integrating sex, age, *Auto-RS*, and metastasis to estimate 1-, 3-, and 5-year overall survival. The points assigned to each variable (top line) are summed into a total score, then mapped to survival probabilities. The inset RCS plot shows spline knot positions (not sequentially aligned by age). (**E**) A calibration plot of CpM. Predicted overall survival (*x*-axis) versus observed overall survival (*y*-axis, Kaplan–Meier). Black dashed line, ideal concordance; blue ×, pointwise calibration; vertical ticks, predicted probability distribution. (**F**) A nomogram of the autophagy prediction model (ApM), integrating *Auto-RS*, age, and sex (excluding metastasis) for 1-, 3-, and 5-year overall survival estimation. Point assignment and total score mapping as in (**D**). (**G**) Calibration plot for ApM, conventions as in (**E**). (**H**) Concordance index (C-index) comparison among CpM, BpM, and ApM. Higher C-index indicates better discrimination. The *p* values were calculated using a likelihood ratio test.

To assess clinical utility, we incorporated *Auto-RS* into prognostic modeling for OS. In univariate analysis, *Auto-RS* showed significant predictive power (hazard ratio = 1.091, 95% CI: 1.047–1.136, *p* < 0.001) (Figure 2A). Multivariate Cox regression confirmed that both metastasis (hazard ratio = 5.340, 95% CI: 2.840–10.042, *p* < 0.001) and *Auto-RS* (hazard ratio = 1.058, 95% CI: 1.012–1.107, *p* < 0.05) were independent predictors of survival (Figure 2B). Time-dependent ROC analysis further identified *Auto-RS* as the most accurate single predictor (AUC = 0.88), outperforming metastasis (0.83), sex (0.45), and age (0.39) (Figure 2C). We next integrated *Auto-RS* into a complete prediction model (CpM), generating individualized nomograms for 1-, 3-, and 5-year survival (Figure 2D) and total point scores for all 139 patients (Table S1). Calibration analysis demonstrated strong agreement between predicted and observed survival, correcting deviations observed in BpM (survival probability: 0.6–1.0) (Figure 2E). Notably, CpM also produced a wider spread of predicted survival probabilities, reflecting improved capture of inter-patient heterogeneity.

Recognizing that clinical metastasis diagnosis is often delayed and survival improvements for metastatic OS have remained stagnant, we developed the autophagy prediction model (ApM), which combines *Auto-RS* with age and sex while deliberately excluding metastasis to enable prognostic assessment. ApM retained strong predictive power with a broad range of individualized survival estimates, demonstrating its ability to capture prognostic heterogeneity independently of metastasis (Figure 2F,G). Although ApM’s C-index (0.709; 95% CI: 0.640–0.778) was slightly lower than that of BpM (0.741; 95% CI: 0.679–0.803) and CpM (0.788; 95% CI: 0.731–0.845), ApM still achieved reliable risk stratification (Figure 2H). Patient-level comparisons across the three models are provided in Table S1. In summary, *Auto-RS* confers independent prognostic value, while ApM demonstrates the feasibility of metastasis-independent prediction, offering a framework for potentially early risk stratification.

### 2.5. Cellular Origin of Auto-RS Signals

**Figure 3:**
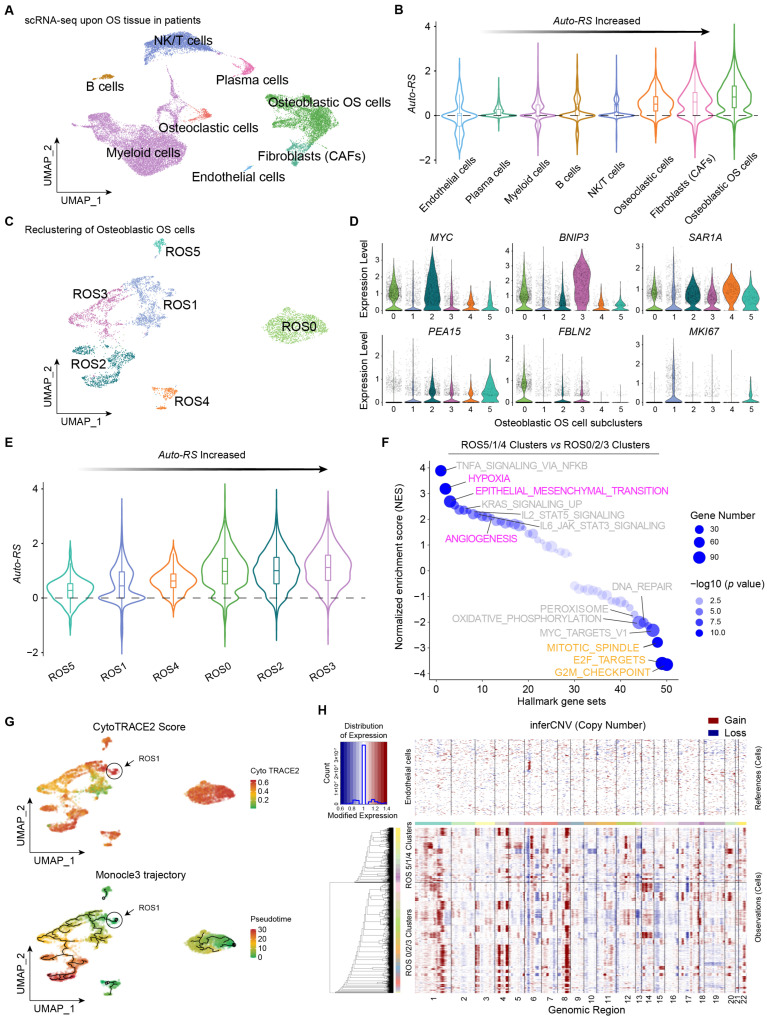
Single-cell analysis reveals the cellular origin and molecular basis of *Auto-RS* prognostic signals. (**A**) A uniform manifold approximation and projection (UMAP) plot embedding 27,719 cells from six primary OS tissues delineating eight major populations. (**B**) *Auto-RS* distribution across the eight major cell populations, increasing gradually from left to right. (**C**) Reclustering of 5988 osteoblastic OS cells identified six subclusters (ROS0 to ROS5). (**D**) Violin plots showing the expression of *Auto-RS*-related genes, including the differential expression of *FBLN2* and *MKI67*. (**E**) The *Auto-RS* signal was mainly enriched in ROS0/2/3. The per-cell *Auto-RS* shown is calculated directly from the four-gene Cox-weighted formula (see Section 4.4); the dashed reference line at zero is a neutral axis without biological threshold meaning, and negative values reflect the contribution of *PEA15* (negative Cox coefficient) rather than an absence of signal. (**F**) GSEA of hallmark pathways contrasting ROS0/2/3 (purple) with ROS5/1/4 (yellow), revealing the enrichment of hypoxia, EMT, angiogenesis, and KRAS signaling versus proliferative signatures (G2M checkpoint, E2F targets, mitotic spindle). Circle size reflects gene counts and color intensity indicates significance. (**G**) CytoTRACE2 and Monocle3 analysis depicting differentiation potential and pseudotime trajectories. High-stemness, undifferentiated ROS1 cells evolve along distinct branches toward metastasis-associated *Auto-RS^high^* ROS2/3. (**H**) Clustering of copy number variation (CNV) profiles inferred from scRNA-seq data for osteoblastic OS cells and endothelial cells. The clusters (dendrogram) primarily reflect ROS0/2/3 and ROS5/1/4 CNVs (colored bar coded). The CNV signal heatmap, normalized against the ‘normal’ cluster defined by endothelial cells, shows chromosome-wise CNV alterations (columns) across individual cells (rows).

To determine what cells mainly deploy the *Auto-RS*, we carried out single-cell RNA-seq (scRNA-seq) analysis on six primary OS tumors [27]. Unsupervised clustering identified eight distinct populations from 27,719 cells, including myeloid cells (49.53%), osteoblastic OS cells (22.33%), NK/T cells (17.92%), cancer-associated fibroblasts (CAFs, 3.3%), plasma cells (2.54%), osteoclastic cells (1.9%), B cells (1.65%), and endothelial cells (0.82%) (Figure 3A and Figure S3A–C). *Auto-RS* signals were enriched in osteoblastic OS cells and CAFs (Figure 3B). To quantify this enrichment beyond the visualization in Figure 3B, we computed the per-cell *Auto-RS* across all cell types in the integrated single-cell dataset. The score differed markedly across populations (Kruskal–Wallis *p* < 2.2 × 10^−16^): the osteoblastic OS cells (mean *Auto-RS* = 0.86, *n* = 6191) and CAFs (0.61, *n* = 915) carried the highest scores and were significantly elevated relative to all other populations pooled (one-sided Wilcoxon rank-sum, both BH-adjusted *p* < 2.2 × 10^−16^), whereas myeloid, NK/T, B, plasma, and endothelial cells clustered in the lower part of the distribution (Figure S9A).

Within 5988 osteoblastic cells, Louvain clustering identified six subpopulations (ROS0-5) with heterogeneous *Auto-RS* expression (Figure 3C and Figure S3D). *Auto-RS* was elevated in ROS0/2/3 relative to ROS5/1/4, reflecting differential expression of the four autophagy genes (Figure 3D,E). Functionally, ROS0/2/3 were characterized by metastatic programs, with enrichment of adhesion molecules such as *FBLN2*, along with hypoxia, EMT, angiogenesis, and KRAS signaling [28] (Figure 3E,F, Table S9). KEGG and GO analyses further highlighted the activation of skeletal development (ossification, bone and cartilage formation), stress-adaptive pathways (hypoxia, unfolded protein response), and canonical oncogenic cascades including TGF-β, MAPK, PI3K-AKT, FoxO, Wnt, HIF-1, and Relaxin. *Auto-RS*^high^ clusters also upregulated stemness signatures, extracellular matrix (ECM)–receptor interactions, and focal adhesion, consistent with enhanced self-renewal and invasive plasticity (Figure S3E–H). By contrast, *Auto-RS*^low^ ROS5/1/4 displayed proliferative states, marked by *MKI67* expression, enrichment of cell cycle and mitotic pathways, and metabolic activation including oxidative phosphorylation and ATP biosynthesis (Figure 3E,F and Figure S4A–D).

Trajectory inference using *CytoTRACE2* [29] and *Monocle3* [30] revealed less-differentiated, highly proliferative ROS1 cells occupy early pseudotime states with low *Auto-RS*, whereas progression toward high *Auto-RS* ROS2/3 coincides with the acquisition of metastasis-associated transcriptional programs (Figure 3G). Early transitions involved stress response and proteostasis genes (*HSPA5*, *HSP90AA1*, *BAG3*, *ATF3*), intermediate stages showed elevated ribosomal protein expression (*RPL30*, *RPL32*, *RPS3A*), and terminal stages activated inflammatory, anti-apoptotic, and cytoskeletal remodeling modules (*FOS*, *JUN*, *MCL1*, *FN1*), defining a coherent high *Auto-RS* metastatic program (Figure S4E).

Finally, inferred CNV [31] analysis revealed that *Auto-RS* differences were largely independent of global chromosomal alterations (Figure 3H). ROS0/2/3 and ROS5/1/4 subpopulations exhibited similar overall CNV burdens (Figure S5A,B), with only *CDK4* amplification in ROS5/1/4 partially explaining their proliferative phenotype. Other recurrent OS CNVs and targetable signaling genes (*MYC*, *PTEN*, *WWOX*, *FLI1*, *NOTCH2*, *PDGFRA*, *VEGF*, *IGF1R*) [32,33] did not correlate with *Auto-RS* stratification (Figure S5C).

Together, these data indicate that *Auto-RS* signals are most strongly enriched in osteoblastic OS cells, with *Auto-RS*^high^ subpopulations exhibiting stress- and metastasis-related programs, while *Auto-RS*^low^ cells are highly proliferative, bioenergetically active states. *Auto-RS* thus captures dynamic cellular states linked to lineage plasticity and metastatic potential beyond genomic alterations.

### 2.6. Auto-RS Permeate the Metastatic OS Microenvironment

**Figure 4:**
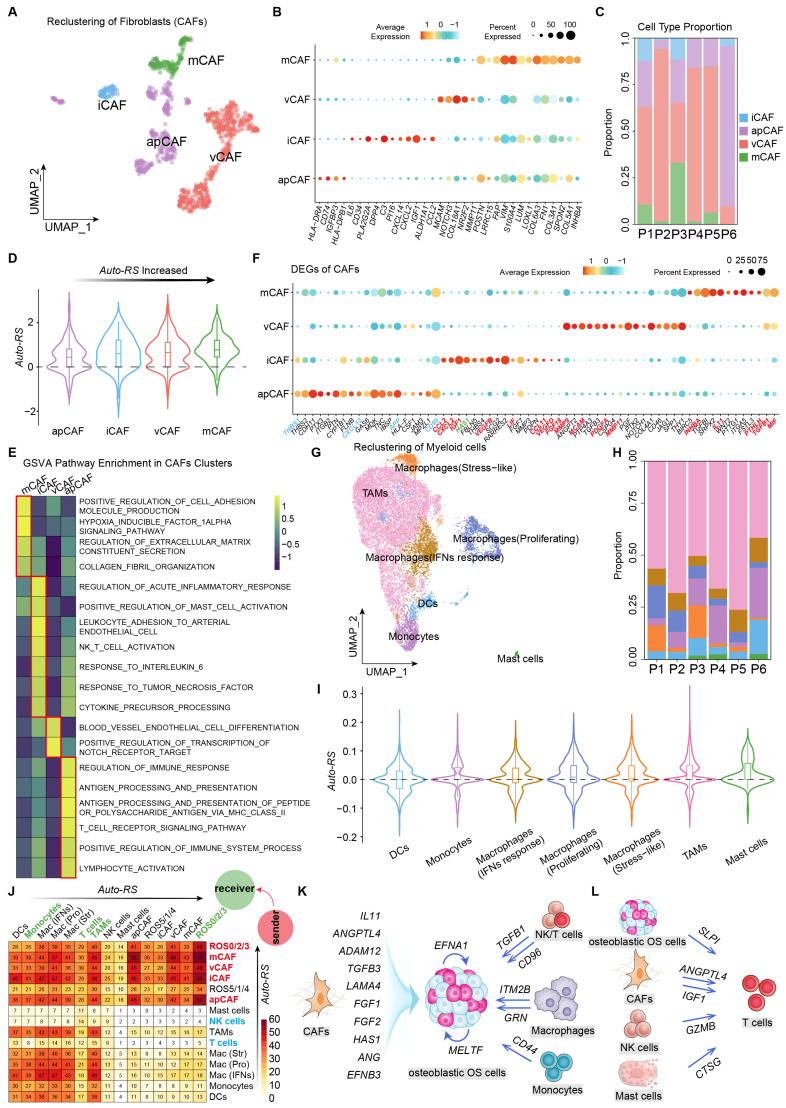
Single-cell dissection of *Auto-RS* signals across CAF and immune populations. (**A**) The reclustering of CAFs revealed four distinct subsets: matrix CAFs (mCAFs), vascular CAFs (vCAFs), inflammatory CAFs (iCAFs), and antigen-presenting CAFs (apCAFs). (**B**) A dot plot of representative marker genes across the CAF subsets. The dot size indicates the proportion of expressing cells; the color intensity reflects relative expression levels. (**C**) A stacked bar chart showing the distribution of CAF subsets across six patients, with vCAFs (48.6%) and apCAFs (31.3%) constituting the majority, followed by iCAFs (14%) and mCAFs (6.1%). (**D**) The distribution of *Auto-RS* values across the CAF subsets, ranked from low to high. (**E**) A heatmap of normalized GSVA scores for selected Gene Ontology pathways in each CAF subset. (**F**) A dot plot showing DEGs across the mCAF, vCAF, iCAF and apCAF clusters. (**G**) The reclustering of myeloid cells identified seven subtypes. (**H**) A stacked bar chart showing the proportion of seven clusters in six OS patients. (**I**) The distribution of *Auto-RS* values across the myeloid subsets, ranked from low to high. (**J**) The number of ligand–receptor interactions among the osteoblastic OS subtypes, CAF subsets, myeloid subsets, NK cells, and T cells. The rows represent sender cells (ordered from low to high *Auto-RS*, bottom to top); the columns represent receiver cells (ordered from low to high *Auto-RS*, left to right). (**K**,**L**) *Auto-RS* identified key pro-metastatic cell–cell communications within the OS microenvironment, including signals promoting osteoblastic OS cell metastasis (**K**) and suppressing T cell antitumor immunity (**L**).

Efficient metastatic progression is tightly orchestrated through dynamic crosstalk among tumor cells, cancer-associated fibroblasts (CAFs), and immune populations [34]. The CAFs exhibited strong *Auto-RS* activity and were segregated into four functional subtypes: matrix CAFs (mCAFs), vascular CAFs (vCAFs), antigen-presenting CAFs (apCAFs), and inflammatory CAFs (iCAFs) [35] (Figure 4A,B). Although the vCAFs and apCAFs were numerically dominant (Figure 4C), the mCAFs contributed the highest *Auto-RS* signal (Figure 4D), characterized by ECM-remodeling and pro-metastatic mediators (*IL11* [36], *TGFB1*, *INHBA*, *MIF*, *PTHLH*). In contrast, the vCAFs were enriched for angiogenesis drivers (*NOTCH1-3*, *JAG1*, *ANGPT2* [37], *PDGFA*, *MMP9/11* [38,39]); the iCAFs secreted inflammatory and chemoresistance-associated cytokines (*IL6*, *LIF*, *IGF1*, *CCL2/11*), with *HAS1* upregulation linked to hyaluronan-mediated metastasis [40,41]; and the apCAFs combined antigen presentation with immune-exclusion programs (*CXCL12*, *THBS1*, *MDK*) (Figure 4E,F, Table S10).

Before analyzing cross-talk, myeloid cells were further resolved into mast cells, dendritic cells (DCs), monocytes, tumor-associated macrophages (TAMs), and three specialized macrophage states (proliferating, stress-like, IFN-responsive) (Figure 4G–I). Notably, neutrophil markers (S100A8/9) localized within the monocyte cluster, precluding neutrophils as a distinct population (Figure S6A,B). The *CellChat* program [42] revealed that osteoblastic OS cells and CAFs with high *Auto-RS* were dominant ‘signal senders’, engaging in dense reciprocal interactions with each other and with immune cells. By contrast, immune populations with low *Auto-RS* contributed relatively few outgoing signals (Figure 4J and Figure S7A, Table S11). The *NicheNet* program [43] highlighted CAF-derived pro-metastatic ligands as key regulators of *Auto-RS*^high^ tumor cells, including *ANGPTL4/ADAM12* (mCAFs), *TGFB3/LAMA4/FGF1* (vCAFs), *FGF2/HAS1/ANG* (iCAFs), and *EFNB3* [44] (apCAFs) (Figure S7B). The ROS0/2/3 cells reinforced this network via autocrine *BMP2/SEMA3A/EFNA1*, with *MELTF* from ROS5/1/4 further augmenting its metastatic potential. Unexpectedly, NK cells, rather than mCAFs, emerged as a major source of *TGFB1*, a canonical EMT driver (Figure S7C). In parallel, NK/T cells transmitted inhibitory signals through *CD96* [45], suppressing their own cytokine responses and cytotoxic activity. TAMs and monocytes further promoted metastasis through *ITM2B/GRN* and *CD44/S100A4/VSTM1/NAMPT*, respectively (Figure 4K).

Although T cells contributed minimally to the *Auto-RS* signal itself, their functional states were sensitively captured. Stratification revealed that *Auto-RS*^high^ T cells received *SLPI* [46] from ROS0/2/3, a serine protease inhibitor that impairs cytotoxic T lymphocyte function by blocking lytic granule release, thereby enhancing tumorigenic and metastatic potential. In addition, mCAFs and iCAFs suppressed T-cell immunity via *ANGPTL4* [47] and *IGF1* [48], respectively. *Auto-RS*^high^ T cells also received proteolytic inhibitory inputs from Mast cells (*CTSG*) and NK cells (*GZMB*), both of which can severely compromise T cell activity (Figure 4L).

Together, these findings delineate a pervasive *Auto-RS* driven signaling network in which CAF subsets and malignant OS cells orchestrate immune suppression and metastatic competence, while *Auto-RS*^high^ immune cells acquire impaired antitumor functionality.

To independently corroborate these single-cell observations at the population scale, we deconvoluted the immune-cell composition of 134 osteosarcoma patients using *CIBERSORT* [49]. The bulk-level patterns were consistent with the single-cell communication networks. *Auto-RS*^high^ tumors showed a relatively immune-sparse microenvironment: CD8^+^ T cell infiltration was lower than in *Auto-RS*^low^ tumors (mean 0.019 vs. 0.040, *p* = 0.027), and M2-polarized macrophages were significantly depleted (0.190 vs. 0.237, *p* = 7.2 × 10^−5^), accompanied by a reciprocal accumulation of undifferentiated M0 macrophages (0.177 vs. 0.146, *p* = 0.041). Together with the single-cell evidence of CAF- and tumor-derived suppressive signaling, these bulk-level patterns (Figure S6C and Table S14) suggest that high *Auto-RS* is associated with an immune-sparse, less-polarized microenvironment. This association indicates that *Auto-RS* may help identify an immune-evasive osteosarcoma subgroup potentially predisposed to immunotherapy resistance, a hypothesis that warrants direct experimental and clinical testing.

### 2.7. Auto-RS Stratification Identifies Therapeutic Vulnerabilities

**Figure 5:**
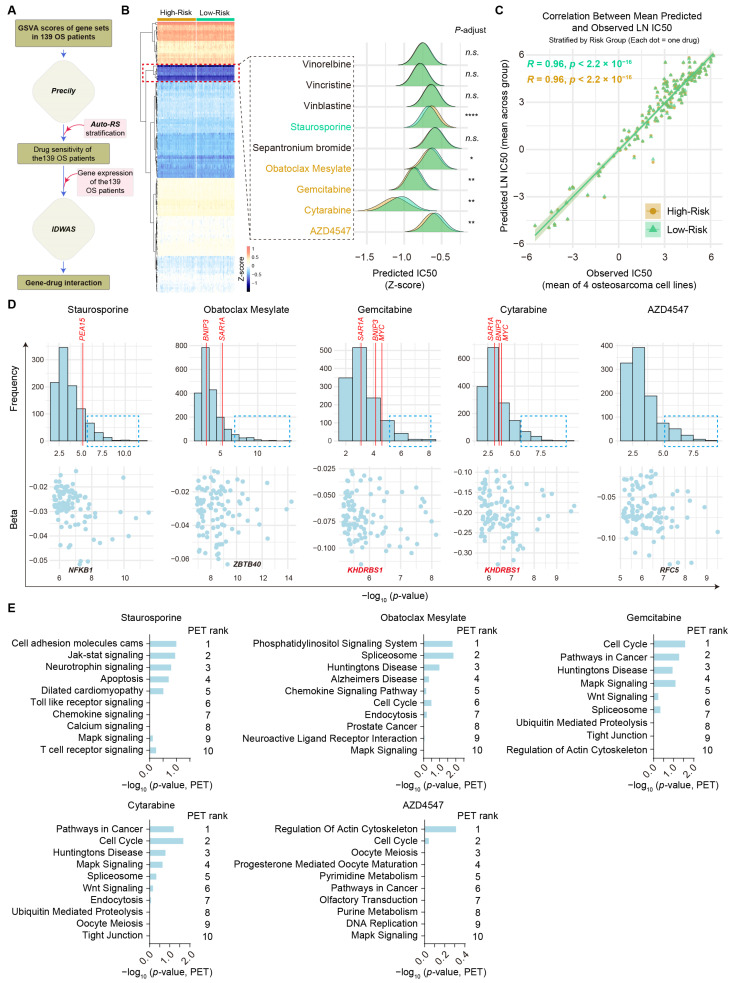
Therapeutic vulnerabilities identified through *Auto-RS*-based drug sensitivity prediction. (**A**) The workflow for predicting drug sensitivity and identifying potential therapeutic targets using *Auto-RS* stratification. (**B**) A heatmap of predicted IC50 Z-scores for 156 compounds across four OS cell lines (HOS, MG63, U2OS, SAOS2) in high- and low-risk groups. Lower IC50 indicates higher predicted sensitivity. Ridge plots show the distribution of predicted IC50 for the nine most sensitive drugs, with green indicating higher sensitivity in low-risk and yellow in high-risk groups. The *p*-values were calculated by Wilcoxon rank-sum test (<0.05 *, <0.01 **, <0.0001 ****, n.s., not significant). (**C**) A scatter plot showing the correlation between the predicted and observed IC50 values (Pearson’s *R*) across high- and low-risk groups. A two-sided *t*-test was used for *p*-value calculation; the shaded areas indicate 95% confidence intervals. (**D**) IDWAS analysis linking patient gene expression profiles to predicted drug response. The top panel shows the frequency of significant gene–drug associations (*p*-values) across all genes. The blue dashed box highlights the top 100 genes with the smallest *p*-values, displayed in the scatter plot below. The beta coefficients indicate the effect of gene expression on the predicted IC50, with more negative values denoting higher sensitivity. The *p*-values were computed using two-sided *t*-tests. (**E**) Pathway enrichment analysis of the target genes corresponding to five predicted sensitive drugs using the Pathway Ensemble Tool (PET). The *p*-values represent combined significance estimated via the Stouffer method integrating GSEA, ORA, and Enrichr results. The top 10 pathways are ranked by average score across methods.

To explore whether *Auto-RS* stratification uncovers druggable dependencies [50], we applied it to *Precily* [51], a deep learning-based pharmacogenomic predictor (Figure 5A). The predicted responses of 139 OS patients to 156 compounds tested across four OS cell lines (HOS, MG63, U2OS, SAOS2) (Table S12) identified nine agents with the highest predicted sensitivity: microtubule-targeting drugs (vinorelbine, vincristine, vinblastine), apoptosis regulators (staurosporine, sepantronium bromide, obatoclax), DNA synthesis inhibitors (gemcitabine, cytarabine), and the FGFR inhibitor AZD4547. In contrast, EGFR/ERBB inhibitors were consistently ineffective, in line with prior reports [52].

Median Z-scores derived from predicted IC50 values identified five compounds with *Auto-RS* specific vulnerabilities: staurosporine showed preferential efficacy in the low-risk group, whereas obatoclax, gemcitabine, cytarabine, and AZD4547 were predicted to show greater sensitivity in the high-risk group (Figure 5B). Notably, gemcitabine and cytarabine are FDA-approved agents for solid tumors and leukemia, respectively. Gemcitabine has demonstrated tolerability in OS combination regimens [53,54], whereas cytarabine has not yet been clinically evaluated in OS, despite preclinical sensitivity in methotrexate-resistant cell lines [55]. The predicted IC50 values correlated strongly with the observed IC50s across GDSC cell lines (Pearson’s *R* = 0.96, *p* < 2.2 × 10^−16^; Figure 5C), supporting the reliability of the predicted sensitivities. As an orthogonal check against independently measured data, both nominated agents ranked among the more potent compounds in the experimentally measured GDSC sensitivity profile of the four OS cell lines (gemcitabine, ninth percentile; cytarabine, 27th percentile of 257 profiled drugs; Figure S9B), indicating they are empirically among the more active agents in OS lines rather than artifacts of in silico prediction. To address the possibility that *Auto-RS* and the drug-sensitivity predictions were derived from the same expression matrix, we repeated the analysis with the risk model and the drug-prediction cohort kept independent: risk was re-assigned in each cohort using the locked *Auto-RS* trained on the other cohort, and predicted sensitivities were re-compared. The principal directions were preserved. Cytarabine and gemcitabine remained predicted as more sensitive in the high-risk group, reaching significance in GSE21257 (*p* = 5.0 × 10^−4^ and *p* = 8.9 × 10^−4^) with a consistent but non-significant trend in TARGET, whereas staurosporine remained more sensitive in the low-risk group (TARGET *p* = 9.5 × 10^−5^). The reproducible direction across independent cohorts, alongside variable per-cohort significance, indicates that the *Auto-RS* drug associations are not an artifact of shared-cohort dependency but should be interpreted as hypothesis-generating (Table S15). Refined imputed drug-wide association (IDWAS) [56] further identified predictive biomarkers, as the number of clinically validated biomarkers for osteosarcoma has been described as ‘strikingly small’ [11] (Figure 5D, and Table S13). Sensitivity to staurosporine in low-risk patients was linked to *NFKB1* expression, whereas *ZBTB40* predicted response to obatoclax. *RFC5* emerged as a marker of AZD4547 sensitivity. For the FDA-approved agents, gemcitabine and cytarabine responses converged on *KHDRBS1* [57] as a shared, highly specific biomarker, consistent with its established role in the chemosensitivity of cancer stem cells. The Pathway Ensemble Tool (PET) [58] highlighted cell cycle regulation as the principal pathway perturbed by gemcitabine and cytarabine (Figure 5E).

Together, these results nominate *Auto-RS* stratification as a hypothesis-generating framework for prioritizing candidate therapeutic vulnerabilities and predictive biomarkers in OS, which will require experimental and clinical validation.

## 3. Discussion

In this study, we asked whether autophagy programs could be leveraged as precise readouts of metastatic biology, offering a paradigm for clinically actionable prognostic modeling in aggressive tumors. The LASSO–Cox regression-based machine learning framework was applied to datasets from 139 patients, integrating autophagy-related transcriptional features with clinical variables to derive the *Auto-RS* signal and construct the ApM. The ApM achieved a C-index of 0.709 for early risk stratification and further enhanced individualized prognostic management when integrated with metastasis information. We ensured that models were trained on the currently most comprehensive clinical and transcriptomic OS datasets and then evaluated across independent single-cell datasets. Confronted with expression landscapes encompassing 13,770 gene profiles, the *Auto-RS* signature distilled metastasis-specific patterns and prioritized a minimal yet informative gene set, which in combination with age and sex, yielded robust prognostic predictions. *Auto-RS* generalized to independent single-cell sequencing data from other laboratories, served as a conserved transcriptional signature capable of penetrating the complex tumor microenvironment, capturing metastatic competence across tumor cell and stromal compartments. This deep conservation of *Auto-RS* suggests that its upstream regulation may represent a process associated with the malignant progression of tumors.

Key innovations underlying *Auto-RS* performance stem from the iterative, survival-supervised screening of autophagy programs within the validation pipeline, combined with independent single-cell functional interpretation. These two components, though distinct, are complementary; the integrated validation approach outperformed single-aspect analysis. A representative case was that models constructed on bulk transcriptomes or conventional metastasis-associated transcripts exhibited limited interpretability in single-cell analyses owing to noisy signal origins (Figure S8). For example, *Auto-RS* appeared to derive predominantly from B cells and plasma cells. Likewise, *Auto-RS* displayed restricted discriminatory power across cellular subpopulations, a result at odds with the marked heterogeneity of the OS microenvironment [59]. Thus, independent interpretability validation proved essential; incorporating both the cellular origins of signals within the tumor microenvironment and single-cell functional information yielded a more comprehensive understanding of prognostic states.

*Auto-RS* delineated the cell-intrinsic functional changes that contributed most to prognostic predictions in OS. We confirmed that osteoblastic OS cells and CAFs, previously reported in the literature as key drivers of metastasis [59], carried the greatest weight in the *Auto-RS* prediction process. This would be consistent with *Auto-RS* learning biologically meaningful transcriptional features rather than simply fitting to dataset-specific expression changes. Unlike studies restricted to a specific cell type [60,61], *Auto-RS* enables pinpointing metastasis-promoting responses unique to each cellular compartment. Prior to extensive experimental validation, *Auto-RS* may thus serve as a guiding framework to simultaneously distinguish critical pro-metastatic cell types and their signaling molecules, facilitating the discovery of therapeutic targets and candidate drugs. Our standardized bioinformatic analyses combined with *Auto-RS* attempted to address these challenges, highlighting several molecular targets (*IL11*, *HAS1*, *ANGPTL4*, *SLPI*, *IGF1*) and the drug cytarabine as candidate vulnerabilities for further investigation.

*Auto-RS* is biologically grounded, reflecting autophagy-mediated transcriptional ‘stress fingerprints’ that are tightly linked to metastasis. Functionally, *BNIP3* promotes anoikis resistance during ECM detachment [62,63], *SAR1A*-mediated secretory autophagy links ER stress to metastasis [64,65], *MYC* drives ER stress-induced autophagy to facilitate malignant transformation [66], whereas *PEA15* exerts tumor-suppressive effects [67,68]. These mechanistic insights indicate that *Auto-RS* captures conserved cellular stress responses within tumor cells rather than random transcriptional noise. Although the roles of these genes in stromal and immune compartments remain less well defined, *Auto-RS* may be best interpreted as a composite readout of stress-adaptive states across the tumor ecosystem, reflecting the metastasis-associated condition rather than representing a direct causal program driven solely by transcriptional dysregulation of these four individual genes.

Our work underscores the value of deep transcriptomic profiling for unlocking prognostic signals, even from limited patient material. Because transcriptional changes often precede phenotypic manifestations, *Auto-RS* may capture metastatic potential more sensitively than conventional physiological readouts. Nevertheless, several limitations remain. It is unclear whether *BNIP3*, *SAR1A*, *MYC*, and *PEA15* directly drive metastasis or instead mark stress-adaptive states associated with metastatic status; the latter interpretation may be more plausible, as it is unlikely that these four genes alone could orchestrate consistent functional transitions across diverse cell types. Beyond this, our study has several methodological limitations. First, all analyses are based on retrospective, publicly archived cohorts, so the prognostic stratification and drug sensitivity predictions are associative and require prospective, ideally multi-institutional, validation before clinical inference. Second, although OS is rare and our integrated discovery set (*n* = 139) is comparable to or larger than many published OS cohorts, the sample size remains modest, which, together with the attenuation of the 5-year time-dependent AUC (0.58–0.72 across external evaluations), may limit statistical power, particularly for median-dichotomized survival comparisons and subgroup analyses; consistent with this, although the continuous *Auto-RS* remained a significant predictor under strict bidirectional transfer between TARGET and GSE21257 and in the independent third cohort (GSE16091, *n* = 34), the Kaplan–Meier comparisons based on median dichotomization did not reach significance in these smaller external cohorts (log-rank *p* = 0.08, 0.15, and 0.417), reflecting limited power rather than an absence of signal. Third, the single-cell analyses are based on six primary tumors, constraining the detection of rare populations and the generalizability of the microenvironmental findings. Fourth, the discovery cohorts were generated on different platforms (microarray for GSE21257 and RNA-seq for TARGET); although batch effects were corrected and each cohort was normalized independently for external validation, residual cross-platform variation cannot be fully excluded, and these cohort-level operations, namely joint batch correction and a population median, cannot be computed for a single prospective patient. Fifth, the drug sensitivity results are computational predictions anchored to GDSC cell line IC50 values; they are hypothesis-generating and assume that cell line responses approximate patient responses, which requires experimental and clinical confirmation. Finally, although OS is predominantly a pediatric and adolescent malignancy, a dedicated pediatric versus adult subgroup analysis was not performed; the predominantly pediatric composition of these cohorts leaves a small adult subgroup with limited power for such a comparison, which we note as a direction for future work.

A further consideration concerns how *Auto-RS* could be translated to the evaluation of an individual prospective patient. The identification of *BNIP3*, *MYC*, *PEA15*, and *SAR1A* as a survival-supervised, biologically grounded autophagy panel stands independently of any particular scoring procedure, and the panel remained stable across resampling and bidirectional external transfers. What the present retrospective data cannot establish is the quantitative calibration needed to score a single patient, namely a normalization reference and an absolute cutoff that do not depend on the composition of a contemporaneous cohort. We therefore regard *Auto-RS* as reported here as a discovery-stage instrument, with the continuous four-gene combination as its primary output and the cohort median as a discovery-stage stratification device rather than a clinical threshold. A deployable single-patient model would require two further steps. First, the four-gene coefficients and the decision threshold should be estimated once on a dedicated prospective, ideally multi-institutional cohort of adequate size with uniform pre-analytic handling and mature follow-up, and then frozen, with the threshold selected by a clinically anchored criterion on that cohort rather than by the median of the cohort under test. Second, measurement should move to a closed, standardized assay that renders each sample self-contained, such as a targeted RT-qPCR or NanoString panel of the four signature genes together with a fixed set of housekeeping or reference genes, or, if sequencing is retained, a targeted RNA-sequencing panel with spike-in reference controls; in each case expression is normalized within the individual sample, for example by a delta-Ct-type internal reference, rather than against a population, and the assay is run under a fixed standard operating procedure with calibrator materials so that scores remain comparable across runs and sites. On such a frozen scale, a single pre-specified cutoff can be applied to one patient at a time, and the locked model would then require analytical validation (precision, reproducibility, and cross-site and cross-platform concordance) followed by prospective clinical validation that the cutoff retains prognostic value, the same staged route taken by established expression-based assays such as Oncotype DX, Prosigna, and MammaPrint. Notwithstanding these limitations, the *Auto-RS* signature provides an interpretable, single-cell-anchored, and externally validated framework for prognostic stratification in osteosarcoma, and a basis for prospectively testable therapeutic hypotheses. These findings should be interpreted in conjunction with other clinical and laboratory assessments.

## 4. Methods

### 4.1. Patient Data Acquisition and Quality Control

RNA sequencing data matrices for TARGET were obtained from the Genomic Data Commons (https://portal.gdc.cancer.gov/) (accessed on 15 March 2025). These matrices integrate both the gene expression profiles and clinical characteristics of osteosarcoma patients. Genes with zero expression were removed, and duplicate genes were merged by averaging their expression values. The processed data were subsequently log2-transformed. Notably, two TARGET cases were excluded due to missing survival data, yielding 86 valid TARGET samples for subsequent analysis. In addition, the expression matrix and corresponding clinical information of the GSE21257 cohort were downloaded from the Gene Expression Omnibus (GEO, https://www.ncbi.nlm.nih.gov/geo/) (accessed on 21 March 2025). Preprocessing followed the same pipeline, including the removal of genes with zero expression and the averaging of duplicate genes. Patients lacking complete clinical information (follow-up time, age, sex, metastasis status, or survival outcome) were excluded. After quality control, the TARGET cohort comprised 86 OS samples with 56,515 genes, whereas the GSE21257 cohort included 53 OS samples with 24,970 genes, resulting in a total of 139 samples across both cohorts. An additional osteosarcoma cohort, GSE16091 (*n* = 34), with overall survival annotation was obtained from GEO and used exclusively for independent external validation.

### 4.2. Autophagy Gene Extraction

A total of 222 autophagy-related genes were obtained from the Human Autophagy Database (HADb, https://www.autophagy.lu/v1/) (accessed on 3 February 2025) (Table S2). Of these, 222 autophagy genes were extracted from the TARGET cohort and 193 from the GSE21257 cohort, with 193 autophagy genes shared across both datasets. The intersection of autophagy genes was visualized using a Venn diagram generated with the R package VennDiagram (v1.7.3).

### 4.3. Batch Effect Correction and Identification of Differentially Expressed Genes

To correct for batch effects, expression matrices from TARGET and GSE21257 were combined and corrected using limma::removeBatchEffect (with the sva package, v3.54.0, loaded in the environment), specifying the dataset of origin as the batch variable and a design matrix (model.matrix) that preserved metastasis status as the biological covariate of interest. In addition, BatchServer (https://lifeinfo.shinyapps.io/batchserver/) (accessed on 10 February 2025), an online tool integrating principal component analysis (PCA) implemented in the factoextra package (v1.0.7), was applied to eliminate and assess batch effects. The results were visualized with pie charts and PCA plots. The merged cohort consisted of 193 autophagy genes (Table S3) across 139 OS samples and was used for subsequent analyses. Differential expression analysis between metastatic and non-metastatic groups was assessed using the limma package (v3.62.2), with the thresholds set as |log2FoldChange| > 0.2 and *p* < 0.05. Heatmaps and volcano plots were generated to visualize DEGs using the R packages pheatmap (v1.0.12), RColorBrewer (v1.1.3), and ggplot2 (v3.5.1).

### 4.4. Construction and Validation of the Prognostic Risk Model

To construct a robust prognostic model, we employed a supervised machine learning framework with iterative resampling and multi-stage model selection. Patient survival labels supervised every step of the procedure; we integrated the expression matrix of DEGs with overall survival data and combined iterative feature selection with a dual-validation mechanism to assess robustness across resampling. To balance feature selection stringency with interpretability, we adopted a hybrid approach that integrates the least absolute shrinkage and selection operator (LASSO) with bidirectional stepwise regression, which optimizes the model by minimizing the AIC.

The iterative procedure was designed as follows:

1. Data Partitioning: (1) The dataset was randomly split into training and testing sets in a 1:1 ratio using the createDataPartition function. (2) Within each run, random splitting was repeated for up to 1000 iterations, and the run terminated (break) at the first split that passed dual-validation in both partitions. This run was independently repeated 20 times, accumulating 20 candidate models (Table S5).
2. Feature Selection: (1) Univariate Cox regression was performed to assess the association between gene expression and overall survival (*p* < 0.05), retaining only those genes that were statistically significant in the training set. (2) LASSO regression: (i) Performed with 10-fold cross-validation to determine the optimal λ value. (ii) Genes with non-zero coefficients at lambda.min were retained. (iii) If no gene was selected, the iteration was skipped.
3. Multivariate Cox Regression Model Construction: (1) The selected features were subjected to bidirectional stepwise regression. (2) The *Auto-RS* was calculated using the following formulation:
  $$\mathrm{Auto}-\mathrm{RS}=\sum_{i=1}^{n}(\beta_{i}\times {Exp}_{i})$$
  where *β_i_* is the Cox regression coefficient and *Exp_i_* is the expression level of gene *i*.
4. Model Validation: (1) Validation was conducted in both the training and testing sets: (i) Survival difference was evaluated using the log-rank test (*p* < 0.05). (ii) ROC curve analyses were performed, and models with AUC > 0.75 were considered acceptable. (2) Within each run, iteration terminated (break) at the first split meeting both criteria; repeating the run 20 times yielded 20 candidate models (Table S5). For each model, samples in both the TARGET and GSE21257 cohorts were divided into high-risk and low-risk groups based on the median Auto-RS for further validation. Groups with statistically significant survival differences (*p* < 0.05) in both cohorts were retained, resulting in 8 validated models (Table S6).

To optimize model complexity and performance, we prioritized models with the best balance of fit and simplicity using stepwise regression and assessed their discrimination ability using the concordance index (C-index). The steps for model selection are as follows:

- Compute the ΔAIC (AIC value minus the minimum AIC across models).
- Exclude models with ΔAIC > 10.
- Among the remaining models, rank them by C-index in descending order and select the one with the highest C-index.

Through this process, the best iteration was ultimately selected as the optimal prognostic model for downstream analyses (Table S7).

For model construction, we used the survival package (v 3.8-3) to perform univariate Cox regression and identify the autophagy gene significantly associated with overall survival (*p* < 0.05). Within each split, LASSO–Cox feature selection was performed with glmnet (v4.1.8) using 10-fold cross-validation to determine λ; up to 1000 splits were evaluated per run until the dual-validation criteria were met. The final multivariate Cox model was built using the ‘coxph’ function combined with stepwise regression to derive the optimal prognostic signature.

Model performance was visualized using Kaplan–Meier survival curves and time-dependent ROC curves were generated using survival (v3.8.3), survminer (v0.5.0), and timeROC (v0.4), enabling the assessment of sensitivity and specificity. A risk heatmap for the four model genes was generated using pheatmap (v 1.0.12). Patient *Auto-RS* distribution plots and survival status scatter plots were generated using ggplot2 (v 3.5.1).

To evaluate whether the *Auto-RS* functioned as an independent prognostic factor, univariate and multivariate Cox regression analyses were performed using the survival package (v3.8-3).

Finally, nomograms were constructed to visualize the prognostic model components. Three types of models were defined:

- Autophagy prediction model (ApM): including *Auto-RS*, age, and sex.
- Basic prediction model (BpM): including metastases, age, and sex.
- Complete prediction model (CpM): including *Auto-RS*, metastases, age, and sex.

In the nomograms, the age variable was modeled using a restricted cubic spline function, with three knots (rcs, k = 3) to capture potential non-linear associations with the outcome variable. Quantified scoring tables were generated Table S1. The rms package (v 7.0.0) was used to compute calibration curves for evaluating prediction reliability—models with calibration curves closer to the diagonal line indicate better predictive accuracy. Additionally, the C-index was calculated to evaluate the predictive ability of each nomogram.

Validation. To evaluate generalizability beyond the internal resampling, we performed strict external validation using a lock-and-transfer design. The four-gene *Auto-RS* panel (*BNIP3*, *MYC*, *PEA15*, *SAR1A*) was treated as a locked signature, and no feature selection was repeated on the validation data. Each cohort was normalized independently (within-cohort z-score), with no joint batch correction between cohorts. Cox coefficients were estimated in the training cohort only, the high/low risk threshold was fixed at the training-cohort median, and the locked model was then applied to the held-out cohort. This procedure was performed bidirectionally (TARGET to GSE21257 and GSE21257 to TARGET). To further assess generalizability in a cohort that never participated in any stage of model construction, the same locked signature was additionally applied to an independent third cohort, GSE16091 (*n* = 34), obtained from the NCBI Gene Expression Omnibus. In all validation cohorts, discrimination was assessed by time-dependent ROC (1-, 3-, and 5-year AUC) and Harrell’s C-index, the continuous *Auto-RS* was tested as a predictor of overall survival by Cox regression (hazard ratio per standard deviation), and median-dichotomized high/low groups were compared by the log-rank test. All external-validation analyses were performed in R using the survival (v3.8-3), timeROC (v0.4), and survminer (v0.5.0) packages.

### 4.5. Single-Cell RNA Sequencing Data of OS Patients

Raw scRNA-seq data of six primary OS tissues were obtained from the GEO database (GSE162454 [27]). Downstream analyses were run with Seurat (v5.2.1) package in R (v4.4.2). For each single-cell RNA-seq data, we created a Seurat object with the CreateSeuratObject function. All Seurat objects were merged into one and filtering steps as follows were applied for the merged data: (1) cells with fewer than 200 detected genes, more than 6000 detected genes, or with UMI counts exceeding 30,000 were excluded; (2) cells with a percentage of mitochondrial UMI count larger than 10% were filtered out; (3) the genes detected in more than 3 cells were retained and all ribosomal genes were filtered out. After quality control, a total of 27,719 high-quality cells were retained. Since the samples of OS tissues come from different patient individuals, this may introduce technical and biological batch effects in downstream analysis. Therefore, based on PCA, the RunHarmony function of harmony (v1.2.3) R package was used to correct the batch effect.

### 4.6. Cell Type Identification and Clustering

Initial sample-level clustering was performed in three sequential steps: (1) dimensionality reduction by principal component analysis (PCA) using all genes as variable features; (2) construction of a shared nearest-neighbor graph in PCA space using the FindNeighbors function; (3) unsupervised clustering using the FindClusters function (unsupervised clustering was performed using the shared nearest neighbor (SNN) modularity optimization algorithm with the Louvain method) at a resolution of 0.2. DEG analysis was performed using the FindAllMarkers function in the Seurat package. Significant DEGs were identified using a cutoff of an FDR-adjusted *p*-value < 0.05 and a log2 fold change > 0.25. Cell type annotation was performed based on canonical marker gene expression, as follows: Myeloid cells: *LYZ*, *CD68*, *CD163*. Cancer-associated fibroblasts (CAFs): *FBLN1*, *ACTA2*, *TAGLN*, *COL3A1*, *COL6A1*. Osteoblastic OS cells: *ALPL*, *RUNX2*, *IBSP*. Osteoclastic cells: *ACP5*, *CTSK*. B cells: *CD79A*, *MS4A1*. NK/T cells: *CD2*, *CD3D*, *CD3E*, *CD3G*, *GNLY*, *NKG7*, *KLRD1*, *KLRB1*. Endothelial cells: *EGFL7*, *PLVAP.* Plasma cells: *IGHG1*, *MZB1*. For sub-clustering, the subset function was used to extract specific cell populations. Reclustering was then performed following the same pipeline for osteoblastic OS cells (resolution = 0.1, *n* = 5988), CAFs (resolution = 0.1, *n* = 915), and myeloid cells (resolution = 0.3, *n* = 12,876).

### 4.7. Pathway Analysis

Gene Ontology (GO) and Kyoto Encyclopedia of Genes and Genomes (KEGG) enrichment analyses of DEGs were performed using the clusterProfiler package (v4.14.6). Gene Set Variation Analysis (GSVA) was conducted with the *GSVA* package (v2.0.7).

### 4.8. Copy Number Variation Analysis

To assess copy number variations (CNVs) in tumor cells across clusters, we performed CNV analysis using the inferCNV package (v1.23.0). Endothelial cells were designated as the reference population, defined as reference Endothelial, to provide a baseline for CNV inference. Comparative analyses were conducted against clusters ROS0/2/3 and ROS5/1/4 to calculate the variability of gene expression intensity across chromosomes in osteoblastic OS cells. The key parameters were set as denoise = TRUE, HMM = FALSE, and cutoff = 0.1, with all other parameters left at their default values. Furthermore, to facilitate interactive exploration of specific genes and chromosomal regions, we utilized the next-generation clustered heatmap (NG-CHM) tool [32]. The processed infercnv.ngchm data can be interactively visualized and explored at https://www.ngchm.net/Downloads/ngChmApp.html (accessed on 16 August 2025).

### 4.9. Prediction of Differentiation States

We used the *CytoTRACE2* [29] package (v1.1.0) to predict the differentiation states of osteoblastic OS cells in our scRNA-seq data. This method provides a continuous assessment of developmental potential through predicted potency scores ranging from 0 to 1, where 0 represents differentiated cells and 1 corresponds to totipotent cells. The raw count matrix was input to generate the prediction using the CytoTRACE2 function, and dedifferentiation scores were plotted with the plotData function using UMAP coordinates generated from Seurat.

### 4.10. Pseudotime Trajectory Analysis

To explore the developmental trajectory of OS cells, we used Monocle3 [30] (v1.4.26) to order cells along a pseudotemporal axis based on transcriptional similarity. A trajectory graph was constructed using the learn_graph function, with the top 1% of cells exhibiting the highest stemness scores in CytoTRACE2 selected as the starting point for pseudotime analysis. To visualize cell distribution and gene expression dynamics along pseudotime, heatmaps of gene expression were generated using the pheatmap package (v1.0.13).

### 4.11. CellChat Analysis

We used CellChat [42] (v1.6.1) for the analysis of cell–cell communication and the analysis followed the official workflow with default parameters unless otherwise specified. First, we loaded the normalized counts into CellChat, followed by the preprocessing steps identifyOverExpressedGenes and identifyOverExpressedInteractions. We then ran the computeCommunProb function for communication analysis with the parameter population.size = FALSE to eliminate possible bias due to cell population size. This resulted in a network of communication strength between all cell states for each of the ligand–receptor pairs that passed the filtering steps. We used the aggregation functions computeCommunProbPathway and aggregateNet to determine the communication strength between cell states at pathway and global levels, respectively. In addition, we applied the NicheNet [43] method to perform a detailed analysis of predicted ligands driving expression changes in target cell subpopulations. Target cell subpopulations of interest included ROS0/2/3 and T cell subsets, while all cells from the relevant sender subpopulations were considered as potential ligand sources. Default parameters were used to predict intercellular interactions.

### 4.12. Drug Response Prediction

We employed the Precily algorithm [51], a machine learning framework for predicting drug responses based on gene expression data. In this approach, pathway enrichment scores derived from RNA-seq expression data via GSVA are integrated with molecular descriptors of drugs (converted from SMILES to vector representations) to form a unified feature matrix. Regression modeling is performed using multiple algorithms, including Random Forests (implemented with the *ranger* package) (v0.18.0), ElasticNet (implemented with the glmnet package), and deep neural networks (implemented with the Keras framework). In the original study, the authors trained and validated the model using CCLE and TCGA datasets, optimized hyperparameters via *k*-fold cross-validation, and ultimately predicted drug sensitivity (IC50 or response classification).

In this study, the data processing pipeline recommended by Precily was followed, and the required input files were prepared as follows:

- Pathway enrichment scores: Based on log_2_ (TPM + 1) gene expression matrices from osteosarcoma samples and cell lines, Gene Set Variation Analysis (GSVA) scores were calculated using the GSVA package (v2.0.7) in R, with min.sz = 5. Gene sets were sourced from the c2 canonical pathway collection in the MSigDB database (MSigDB.CP.v6.1), comprising 1329 gene sets, consistent with the original model training set.
- Drug and sample metadata: Metadata files were initially provided by the authors, and we supplemented them with annotations for osteosarcoma cell lines (HOS, MG63, U2OS, and SAOS2) based on the Genomics of Drug Sensitivity in Cancer (GDSC) database. The two input files were then passed to the Precily drugPred function as follows: drugPred (enrichment.scores, metadata, “OS”). This process generated predicted drug sensitivities (IC50 values) for 139 osteosarcoma patient samples and 4 osteosarcoma cell lines across 156 drugs.

### 4.13. Prediction of Potential Drug Biomarkers

To discover potential biomarkers of drug sensitivity, we utilized the imputed drug-wide association study (IDWAS) method [56]. This approach employs linear ridge regression to identify associations between predicted drug sensitivity and gene expression data. We integrated the predicted drug response data with the gene expression matrix of 139 osteosarcoma samples to systematically evaluate gene–drug correlations.

### 4.14. Pathway Prediction for Potential Drug Biomarkers

To further investigate the biological pathways potentially involved in drug biomarker genes, we employed the Pathway Ensemble Tool (PET) [58]. PET integrates three widely used pathway enrichment approaches (GSEA, ORA, and Enrichr) to calculate independent enrichment scores for each pathway and subsequently generates a consensus ranking across methods. We then derived the average rank across the three approaches and applied the Stouffer method to estimate a combined *p-*value. Finally, the pathways were ranked according to their aggregated scores, enabling the identification of biological mechanisms potentially associated with drug sensitivity.

### 4.15. Immune-Cell Deconvolution by CIBERSORT

To estimate the relative abundance of immune-cell subsets across the OS cohort, we applied *CIBERSORT* [49] to the integrated bulk expression matrix (TARGET and GSE21257, *n* = 139). The LM22 leukocyte signature matrix, which resolves 22 human hematopoietic cell phenotypes, was used as the reference. Deconvolution was performed with 1000 permutations to estimate per-sample statistical significance, and samples with a CIBERSORT permutation *p*-value > 0.05, indicating unreliable deconvolution given the input signal, were excluded from downstream analyses, leaving 134 patients with reliable immune-composition estimates. Per-cell-type fractions were then compared between the *Auto-RS*^high^ and *Auto-RS*^low^ groups using two-sided Mann–Whitney U tests, with Benjamini–Hochberg false-discovery-rate adjustment across the 22 cell types. The relationship between CD8+ T-cell fraction and *Auto-RS* group was additionally examined by Spearman rank correlation.

### 4.16. Statistics

No statistical method was used to predetermine sample size. All statistical analyses were performed in R (v4.4.1). Survival curves were generated using the Kaplan–Meier method and compared with the log-rank test. Comparisons between the two groups were conducted using either the unpaired two-tailed Student’s *t*-test or the Wilcoxon rank-sum test, as appropriate. The likelihood ratio test was employed to assess the statistical significance of differences in c-index between models. The Wald test was used to test the significance of univariate and multivariate Cox models. Pearson and Spearman correlation coefficients were used to evaluate associations between continuous variables. *p* values less than 0.05 were considered statistically significant.

## Figures and Tables

**Table 1:** Clinical and pathological characteristics of patients and their tumors.

Characteristic	TARGET (*n* = 86) No. (%)	GSE21257 (*n* = 53) No. (%)
Diagnosis	Primary osteosarcoma	Primary osteosarcoma
Age (years)		
≤15	46 (53.5)	21 (39.6)
16–20	29 (33.7)	21 (39.6)
>20	11 (12.8)	11 (20.8)
Gender		
Female	37 (43.0)	19 (35.8)
Male	49 (57.0)	34 (64.2)
Metastasis		
Yes	21 (24.4)	34 (64.2)
No	65 (75.6)	19 (35.8)
Tumor Grade		
1	–	13 (24.5)
2	–	16 (30.2)
3	–	13 (24.5)
4	–	5 (9.4)
Unknown	–	6 (11.4)
Histological Subtype		
Osteoblastic	–	32 (60.4)
Fibroblastic	–	5 (9.4)
Chondroblastic	–	6 (11.4)
Other	–	10 (18.8)
Race		
Asian	7 (8.1)	–
Black	7 (8.1)	–
White	51 (59.3)	–
Unknown	21 (24.5)	–
survival time (years)		
≤1	8 (9.3)	4 (7.5)
1–3	33 (38.4)	19 (35.8)
3–5	17 (19.8)	10 (18.9)
≥5	28 (32.5)	20 (37.8)
survival status		
Dead	29 (33.7)	22 (41.5)
Alive	57 (66.3)	31 (58.5)
Sample Sourceand Institution	The tumor tissues used in this study were collected from patients enrolled in Children’s Oncology Group (COG) biology studies and clinical trials and through collaborations with The Hospital for Sick Children (SickKids) in Toronto(Ontario, Canada), Chiba Cancer Center (Chiba, Japan), and the Pediatric Oncology Institute (Sao Paolo, Brazil).	Osteosarcoma pre-chemotherapy biopsy: Centre for Molecular Medicine Norway
Data sources	Gene Chip^®^ Human Exon ST Array (Affymetrix, Santa Clara, CA, USA) https://portal.gdc.cancer.gov/analysis_page?app=CohortBuilder&tab=available_data (accessed on 15 March 2025)	Illumina human-6 v2.0 expression beadchip (San Diego, CA, USA) https://www.ncbi.nlm.nih.gov/geo/query/acc.cgi?acc=GPL10295 (accessed on 21 March 2025)

## Data Availability

All datasets used in this study are publicly available. Bulk RNA-seq data from the TARGET osteosarcoma cohort were obtained through the Genomic Data Commons (GDC, https://portal.gdc.cancer.gov/projects/TARGET-OS) (accessed on 15 March 2025) under project ID TARGET-OS. RNA-seq profiles and clinical infor-mation for the GSE21257 cohort and raw single-cell RNA-seq data for GSE162454 (six primary osteosarcoma tissues) were downloaded from the NCBI Gene Expression Omnibus under accession numbers GSE21257 (https://www.ncbi.nlm.nih.gov/geo/query/acc.cgi?acc=GSE21257) (accessed on 21 March 2025) and GSE162454 (https://www.ncbi.nlm.nih.gov/geo/query/acc.cgi?acc=GSE162454) (accessed on 16 May 2025), the GSE16091 cohort used for independent external validation is likewise available from GEO (https://www.ncbi.nlm.nih.gov/geo/query/acc.cgi?acc=GSE16091) (accessed on 12 June 2026), respectively. All datasets are fully de-identified and publicly accessible without restrictions; no data-use agreements or controlled-access procedures apply. All processed expression matrices generated during this study (including quality-controlled TARGET, GSE21257, and GSE162454 expression files, as well as autophagy-related gene subsets) and the train/test partition tables are openly available in the GitHub repository (https://github.com/Lqz-DC/Autophagy-in-osteosarcoma.git) (accessed on 15 June 2026). No clinical trial data were generated or analyzed, and therefore no individual participant data sharing statements apply. All code necessary for the analyses is available without access restrictions via GitHub at: https://github.com/Lqz-DC/Autophagy-in-osteosarcoma.git (accessed on 15 June 2026).

## Supplementary Materials

The following supporting information can be downloaded at https://www.mdpi.com/article/10.3390/genes17070737/s1, Figure S1: Autophagy gene dysregulation in OS; Figure S2: Auto-RS captures heterogeneity beyond clinical metastasis; Figure S3: Comprehensive characterization of osteoblastic OS cell subclusters; Figure S4: Comprehensive analysis of functional enrichment, interaction networks, and pseudotime expression in osteoblastic osteosarcoma; Figure S5: Relationship between Auto-RS and CNV in osteoblastic OS cells; Figure S6: Marker gene expression patterns of myeloid cell subtypes and immune-cell composition across Auto-RS; Figure S7: Cell–cell communication analysis in the osteosarcoma microenvironment; Figure S8: Model analysis of genes enriched in the whole transcriptome and tumor-related metastasis pathways; Figure S9: Per cell type Auto-RS enrichment, GDSC sensitivity ranking of nominated agents, and strict external validation of the Auto-RS signature; Table S1: Comparison of three models based on nomograms for predicting survival rate; Table S2. Autophagy genes downloaded from Human Autophagy Database; Table S3. Common autophagy related genes (ARGs) both in TARGET and GSE21257 data sets; Table S4. Training and Testing groups randomly divided from 139 OS samples; Table S5: The 20 groups obtained through the iteration process; Table S6. The groups that were significant (*p* < 0.05) in the validation set during 20 cycles; Table S7. Filtering the optimal grouping based on the calculation formula; Table S8: Risk composition classified based on the median value of Auto-RS; Table S9: Enrichment pathways between ROS0/2/3 and ROS1/4/5 based on 50 hallmark gene sets; Table S10: CAFs gsva_c5 go biological process result; Table S11: The interaction between ligands and receptors on cells; Table S12: Drug sensitivity prediction for 139 samples; Table S13: The biomarkers predicted by IDWAS; Table S14: Significant statistical table of immune cells; Table S15: The drug prediction results of the independent cohort.
