# Supplementary material for: An Auto-RS Signature for Prognostic Stratification and Drug Sensitivity Prediction in Osteosarcoma
Source: Genes (Basel). 2026 Jun 26;17(7):737. doi: 10.3390/genes17070737 (PMC13408425; doi:10.3390/genes17070737)
Supplement: Supplementary file 1 [file genes-17-00737-s001.zip › Data Supplement.pdf]

Data Supplement

An Auto-RS Signature for Prognostic Stratification and Drug Sensitivity Prediction in Osteosarcoma

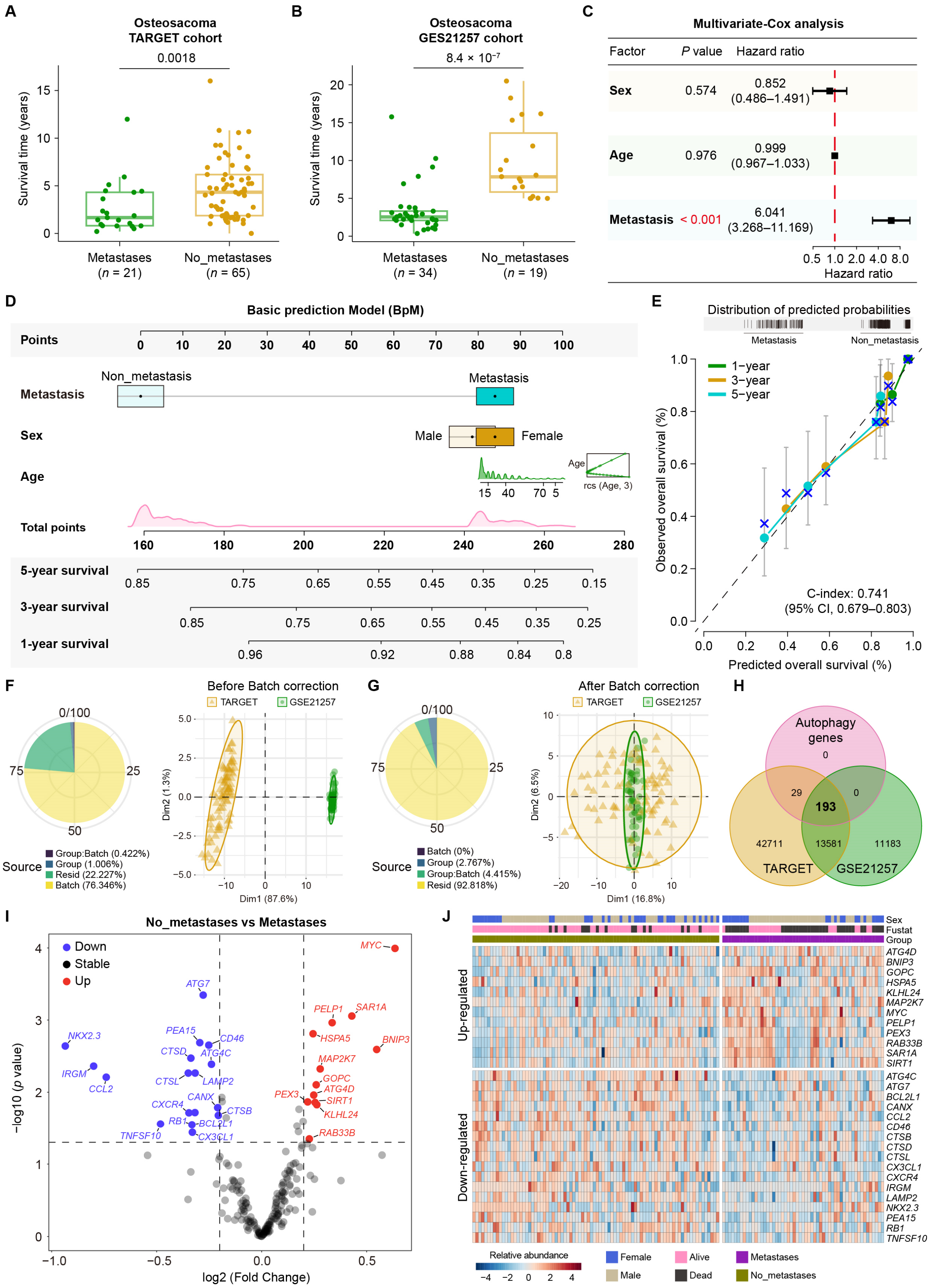
A
Osteosacoma
TARGET cohort
0.0018
Survival time (years)
Metastases
(n = 21)
No_metastases
(n = 65)
B
Osteosacoma
GES21257 cohort
8.4 × 10⁻⁷
Survival time (years)
Metastases
(n = 34)
No_metastases
(n = 19)
C
Multivariate-Cox analysis
Factor
P value
Hazard ratio
Sex
0.574
0.852
(0.486–1.491)
Age
0.976
0.999
(0.967–1.033)
Metastasis
< 0.001
6.041
(3.268–11.169)
0.5 1.0 2.0 4.0 8.0
Hazard ratio
D
Basic prediction Model (BpM)
Points
Metastasis
Non_metastasis
Metastasis
Sex
Male
Female
Age
rcs (Age, 3)
Total points
5-year survival
3-year survival
1-year survival
E
Distribution of predicted probabilities
Metastasis
Non_metastasis
1-year
3-year
5-year
Observed overall survival (%)
C-index: 0.741
(95% CI, 0.679–0.803)
Predicted overall survival (%)
F
Before Batch correction
TARGET
GSE21257
Source
Group:Batch (0.422%)
Group (1.006%)
Resid (22.227%)
Batch (76.346%)
Dim1 (87.6%)
Dim2 (1.3%)
G
After Batch correction
TARGET
GSE21257
Source
Batch (0%)
Group (2.767%)
Group:Batch (4.415%)
Resid (92.818%)
Dim1 (16.8%)
Dim2 (6.5%)
H
Autophagy
genes
0
29
193
0
42711
13581
11183
TARGET
GSE21257
I
No_metastases vs Metastases
Down
Stable
Up
−log10 (p value)
log2 (Fold Change)
MYC
SAR1A
PELP1
HSPA5
BNIP3
MAP2K7
GOPC
ATG4D
PEX3
SIRT1
KLHL24
RAB33B
ATG7
NKX2.3
PEA15
CD46
CTSD
ATG4C
IRGM
CCL2
CTSL
LAMP2
CANX
CXCR4
CTSB
RB1
BCL2L1
TNFSF10
CX3CL1
J
Sex
Fustat
Group
Up-regulated
Down-regulated
Relative abundance
Female
Male
Alive
Dead
Metastases
No_metastases

Figure S1: Autophagy gene dysregulation in OS.

A,B, Survival analyses of the TARGET and GSE21257 cohorts, showing shorter survival time in metastatic patients compared to non-metastatic patients. *P* values were calculated using Wilcoxon rank-sum test.

C, Multivariate COX analyses of OS survival based on sex, age, and metastatic status. Hazard ratio > 1 indicates risk factors, < 1 indicates protective factors. The *P* values for each variable were derived from the Wald test.

D, Overview of the contributions of sex, age, and metastatic status as integrated into the basic prediction model (BpM) and visualized in the nomogram. Each clinical variable was assigned a specific number of points as indicated on the topmost line of the nomogram. The points for all variables were summed to generate a total score, which was then mapped to the probabilities of 1-, 3-, and 5-year overall survival. The knot values displayed in the inset RCS plot correspond to the positions selected for spline fitting; note that they are not arranged sequentially by age along the axis.

E, Calibration plots for the validation of the BpM. The predicted probability of overall survival (x-axis, as estimated by the nomogram) is plotted against the observed overall survival (y-axis, based on Kaplan–Meier estimates). Vertical grey lines indicate the 95% confidence intervals. The black dashed diagonal line represents the ideal reference line, where predictions perfectly match observations. Blue "×" marks represent pointwise calibration results, and short vertical ticks at the top denote the distribution of predicted probabilities across individual samples.

F,G, Variance decomposition (pie charts) and principal component analysis (PCA) of the TARGET (yellow) and GSE21257(green) cohorts shown before (F) and after (G) batch effect correction. Variance was attributed to Group (metastasis vs non-metastasis), Batch (cohort differences), Group: Batch (interaction), and Resid (unexplained). PCA plots show distinct separation between cohorts before correction and improved integration after batch effect removal.

H, Venn diagram illustrating the overlap of autophagy genes, curated from the Human Autophagy Database, and those detected in the TARGET and GSE21257 transcriptional cohorts.

I, The changes in autophagy gene expression in the integrated cohorts comparing metastatic and non-metastatic group.

J, Abundance trajectories of all 29 changed autophagy genes were normalized by row-wise Z-score across samples and clustered according to metastatic status. The distribution of sex and fustat (survival status) among 139 OS patients is shown horizontally above.

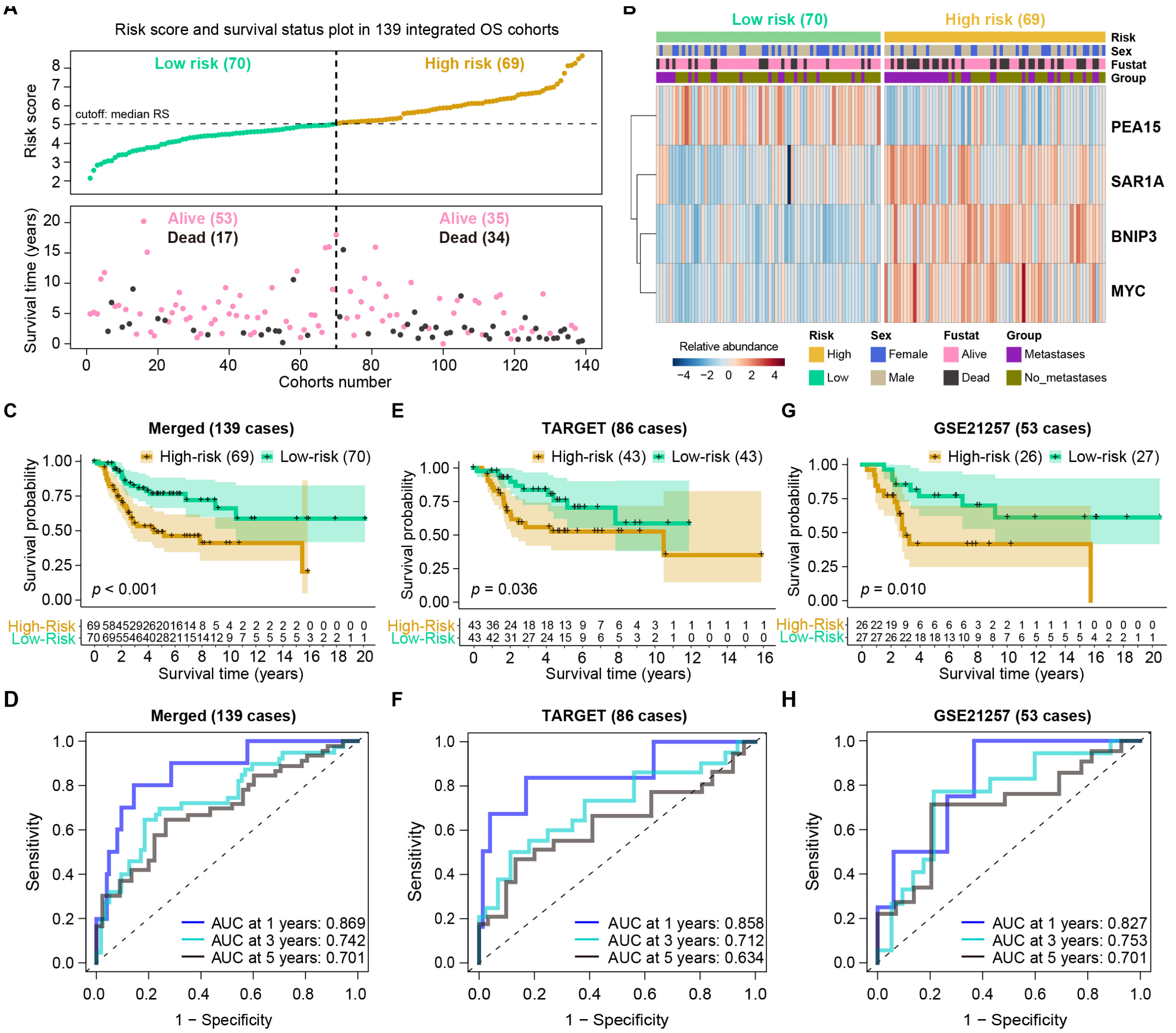

Figure S2: *Auto-RS* captures heterogeneity beyond clinical metastasis

A, Distribution of *Auto-RS* and survival status for all 139 OS patients. Low- and high-risk groups are shown in green and yellow, respectively; alive and deceased patients are shown in pink and black.

B, Heatmap of the four *Auto-RS* component genes, row-wise Z-score normalized across samples and grouped by *Auto-RS*. sex, metastatic status, and survival status (Fustat) are indicated above the heatmap.

C,**E**,**G**, Kaplan–Meier survival curves for the combined cohort (*n* = 139), TARGET cohort (*n* = 86), and GSE21257 cohort (*n* = 53). High-risk patients exhibited significantly shorter survival compared with low-risk patients. *P*-values were calculated using the log-rank test; $P < 0.05$ was considered significant.

D,**F**,**H**, Time-dependent ROC curves for the three cohorts. Blue, cyan, and light gray lines represent 1-, 3-, and 5-year survival predictions, respectively. AUC values reflect model performance, with higher values indicating better predictive accuracy.

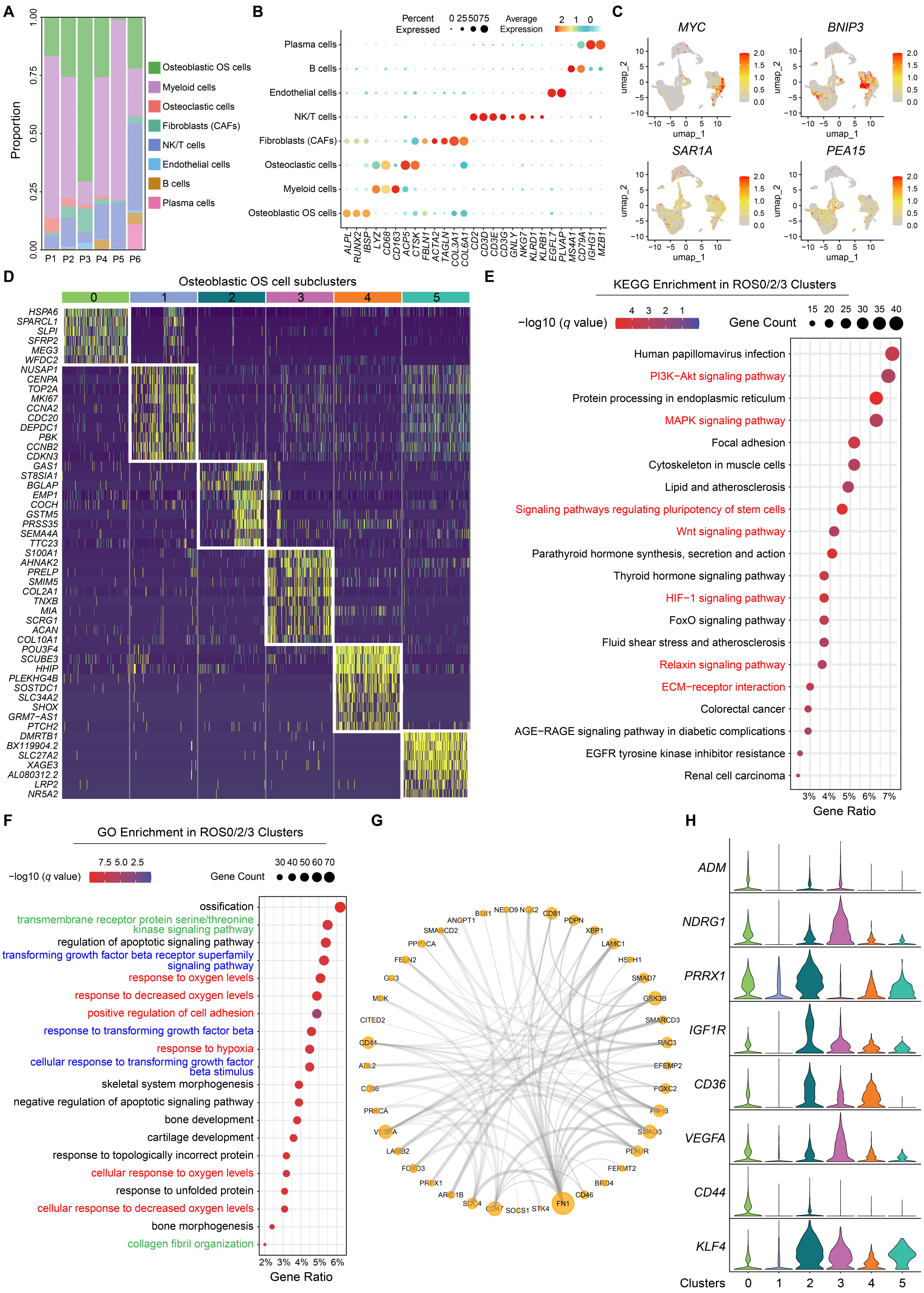
A
Proportion
Osteoblastic OS cells
Myeloid cells
Osteoclastic cells
Fibroblasts (CAFs)
NK/T cells
Endothelial cells
B cells
Plasma cells
B
Percent Expressed
Average Expression
C
MYC
BNIP3
SAR1A
PEA15
D
Osteoblastic OS cell subclusters
E
KEGG Enrichment in ROS0/2/3 Clusters
−log10 (q value)
Gene Count
Human papillomavirus infection
PI3K−Akt signaling pathway
Protein processing in endoplasmic reticulum
MAPK signaling pathway
Focal adhesion
Cytoskeleton in muscle cells
Lipid and atherosclerosis
Signaling pathways regulating pluripotency of stem cells
Wnt signaling pathway
Parathyroid hormone synthesis, secretion and action
Thyroid hormone signaling pathway
HIF−1 signaling pathway
FoxO signaling pathway
Fluid shear stress and atherosclerosis
Relaxin signaling pathway
ECM−receptor interaction
Colorectal cancer
AGE−RAGE signaling pathway in diabetic complications
EGFR tyrosine kinase inhibitor resistance
Renal cell carcinoma
Gene Ratio
F
GO Enrichment in ROS0/2/3 Clusters
−log10 (q value)
Gene Count
ossification
transmembrane receptor protein serine/threonine kinase signaling pathway
regulation of apoptotic signaling pathway
transforming growth factor beta receptor superfamily signaling pathway
response to oxygen levels
response to decreased oxygen levels
positive regulation of cell adhesion
response to transforming growth factor beta
response to hypoxia
cellular response to transforming growth factor beta stimulus
skeletal system morphogenesis
negative regulation of apoptotic signaling pathway
bone development
cartilage development
response to topologically incorrect protein
cellular response to oxygen levels
response to unfolded protein
cellular response to decreased oxygen levels
bone morphogenesis
collagen fibril organization
Gene Ratio
G
H
ADM
NDRG1
PRRX1
IGF1R
CD36
VEGFA
CD44
KLF4
Clusters

Figure S3: **Comprehensive characterization of osteoblastic OS cell subclusters.**

A, Stacked bar chart of OS cluster cell proportions in six patients.

B, Dot plot of representative marker genes across eight cell subtypes. Dot size indicates the proportion of expressing cells; color reflects relative expression.

C, UMAP visualization of the four model genes.

D, Heatmap of the top ten marker genes in the osteoblastic OS subtype.

E, KEGG enrichment of genes upregulated in ROS0/2/3 clusters (top 20 pathways). Dot size shows the number of mapped genes; color indicates significance.

F, GO enrichment of genes upregulated in ROS0/2/3 clusters (top 20 terms). Dot size shows the number of mapped genes; color indicates significance.

G, Protein–protein interaction network of genes in the "positive regulation of cell adhesion" GO term.

H, Violin plot of metastasis-associated genes in the osteoblastic OS subtype.

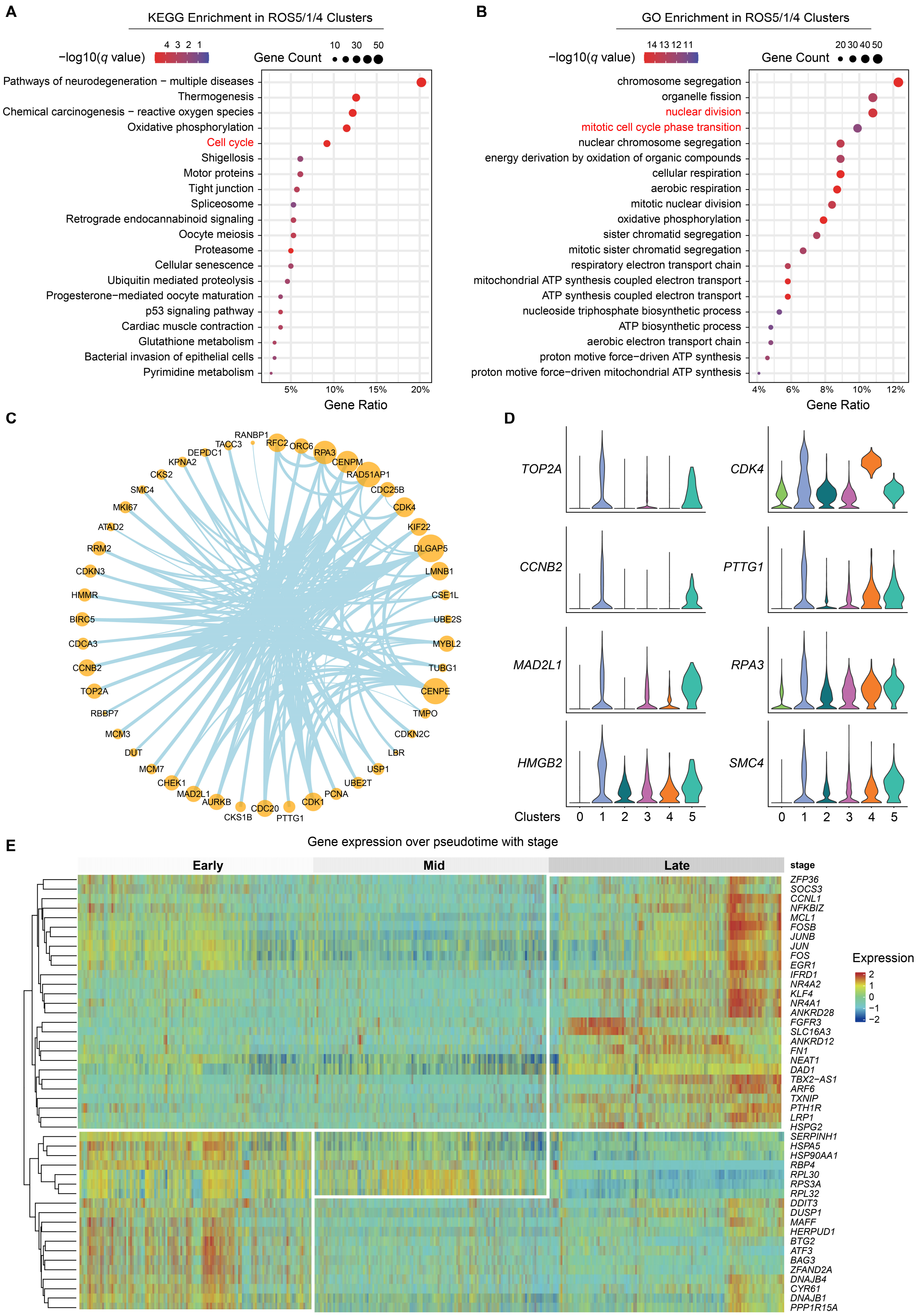
A
KEGG Enrichment in ROS5/1/4 Clusters
−log10(q value)
Gene Count
Pathways of neurodegeneration − multiple diseases
Thermogenesis
Chemical carcinogenesis − reactive oxygen species
Oxidative phosphorylation
Cell cycle
Shigellosis
Motor proteins
Tight junction
Spliceosome
Retrograde endocannabinoid signaling
Oocyte meiosis
Proteasome
Cellular senescence
Ubiquitin mediated proteolysis
Progesterone−mediated oocyte maturation
p53 signaling pathway
Cardiac muscle contraction
Glutathione metabolism
Bacterial invasion of epithelial cells
Pyrimidine metabolism
Gene Ratio
B
GO Enrichment in ROS5/1/4 Clusters
−log10(q value)
Gene Count
chromosome segregation
organelle fission
nuclear division
mitotic cell cycle phase transition
nuclear chromosome segregation
energy derivation by oxidation of organic compounds
cellular respiration
aerobic respiration
mitotic nuclear division
oxidative phosphorylation
sister chromatid segregation
mitotic sister chromatid segregation
respiratory electron transport chain
mitochondrial ATP synthesis coupled electron transport
ATP synthesis coupled electron transport
nucleoside triphosphate biosynthetic process
ATP biosynthetic process
aerobic electron transport chain
proton motive force−driven ATP synthesis
proton motive force−driven mitochondrial ATP synthesis
Gene Ratio
C
RANBP1 TACC3 DEPDC1 KPNA2 CKS2 SMC4 MKI67 ATAD2 RRM2 CDKN3 HMMR BIRC5 CDCA3 CCNB2 TOP2A RBBP7 MCM3 DUT MCM7 CHEK1 MAD2L1 AURKB CKS1B CDC20 PTTG1 CDK1 PCNA UBE2T USP1 LBR CDKN2C TMPO CENPE TUBG1 MYBL2 UBE2S CSE1L LMNB1 DLGAP5 KIF22 CDK4 CDC25B RAD51AP1 CENPM RPA3 ORC6 RFC2
D
TOP2A
CDK4
CCNB2
PTTG1
MAD2L1
RPA3
HMGB2
SMC4
Clusters
E
Gene expression over pseudotime with stage
Early
Mid
Late
stage
Expression
ZFP36 SOCS3 CCNL1 NFKBIZ MCL1 FOSB JUNB JUN FOS EGR1 IFRD1 NR4A2 KLF4 NR4A1 ANKRD28 FGFR3 SLC16A3 ANKRD12 FN1 NEAT1 DAD1 TBX2−AS1 ARF6 TXNIP PTH1R LRP1 HSPG2 SERPINH1 HSPA5 HSP90AA1 RBP4 RPL30 RPS3A RPL32 DDIT3 DUSP1 MAFF HERPUD1 BTG2 ATF3 BAG3 ZFAND2A DNAJB4 CYR61 DNAJB1 PPP1R15A

Figure S4: Comprehensive analysis of functional enrichment, interaction networks, and pseudotime expression in osteoblastic osteosarcoma.

A, KEGG enrichment of genes clusters (top 20 pathways). Dot size shows the number upregulated in ROS5/1/4 of mapped genes; color Indicates significance.

B, GO enrichment of genes upregulated in ROS5/1/4 clusters (top 20 terms). Dot size shows the number of mapped genes; color indicates significance.

C, Protein–protein interaction network of genes in the "cell cycle" GO term.

D, Violin plot of proliferation-associated genes in the osteoblastic OS subtype.

E, Heatmap of pseudotime dynamics of representative differentially expressed genes. Rows are genes, columns are cells; color indicates scaled expression. Top annotation denotes pseudotime stages (Early, Mid, Late).

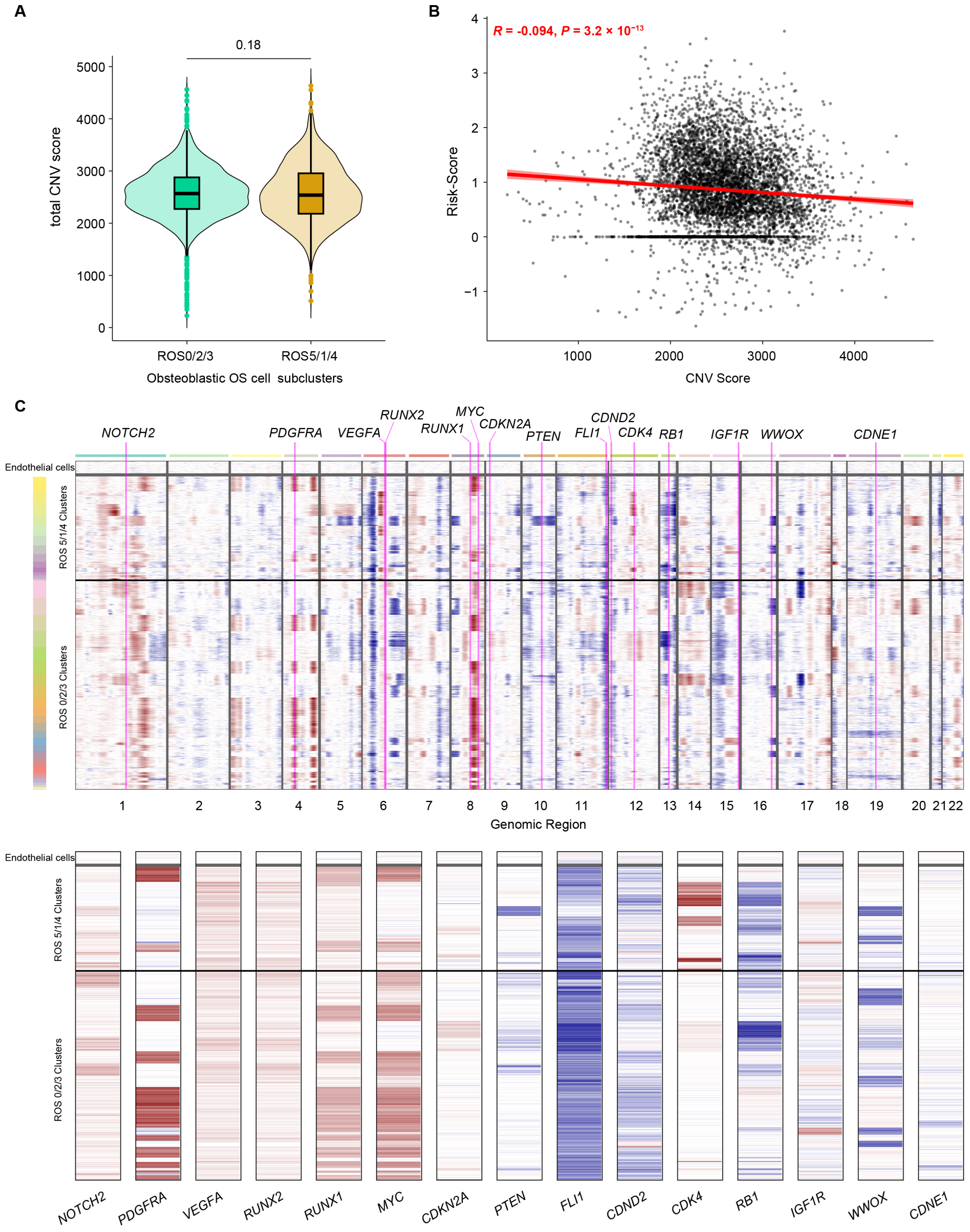
A
0.18
total CNV score
5000
4000
3000
2000
1000
0
ROS0/2/3
ROS5/1/4
Obsteoblastic OS cell subclusters
B
R = -0.094, P = 3.2 × 10⁻¹³
Risk-Score
CNV Score
C
NOTCH2
PDGFRA
VEGFA
RUNX2
RUNX1
MYC
CDKN2A
PTEN
FLI1
CDND2
CDK4
RB1
IGF1R
WWOX
CDNE1
Endothelial cells
ROS 5/1/4 Clusters
ROS 0/2/3 Clusters
Genomic Region

Figure S5: Relationship between *Auto-RS* and CNV in osteoblastic OS cells

A, Total CNV scores compared between ROS0/2/3 and ROS5/1/4 clusters (Wilcoxon rank-sum test).

B, Correlation between CNV scores and *Auto-RS* assessed by Spearman's rank correlation.

C, Next-generation clustered heatmap (NG-CHM). Pink lines mark selected chromosomal segments; the lower panel shows a magnified view of the corresponding region.

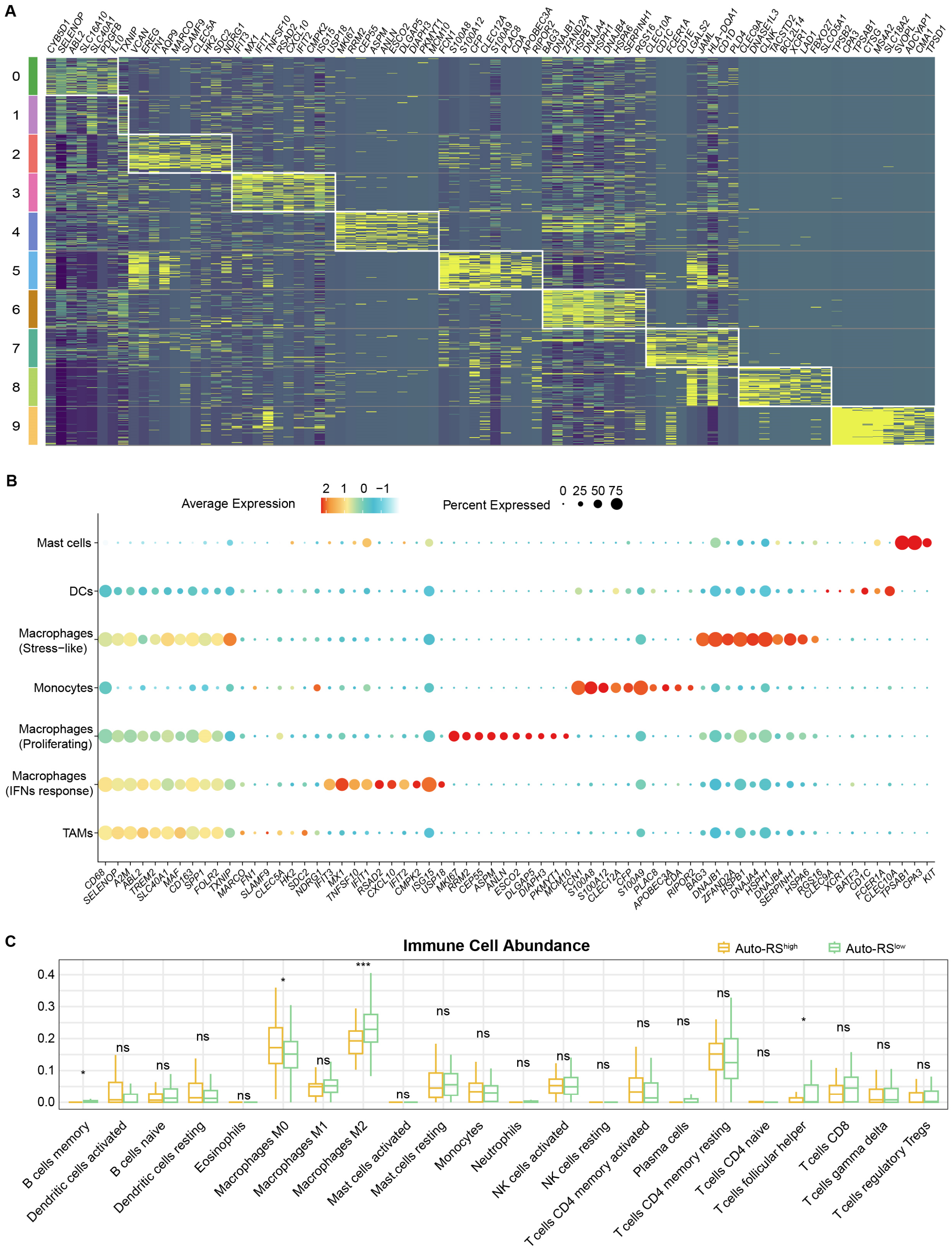
A
0
1
2
3
4
5
6
7
8
9
B
Average Expression
2 1 0 −1
Percent Expressed
0 25 50 75
Mast cells
DCs
Macrophages (Stress−like)
Monocytes
Macrophages (Proliferating)
Macrophages (IFNs response)
TAMs
C
Immune Cell Abundance
Auto-RS^high
Auto-RS^low
0.4
0.3
0.2
0.1
0.0
B cells memory
Dendritic cells activated
B cells naive
Dendritic cells resting
Eosinophils
Macrophages M0
Macrophages M1
Macrophages M2
Mast cells activated
Mast cells resting
Monocytes
Neutrophils
NK cells activated
NK cells resting
T cells CD4 memory activated
Plasma cells
T cells CD4 memory resting
T cells CD4 naive
T cells follicular helper
T cells CD8
T cells gamma delta
T cells regulatory Tregs

Figure S6: Marker gene expression patterns of myeloid cell subtypes and immune-cell composition across *Auto-RS*.

A, Heatmap of the top ten marker genes in myeloid subtypes.

B, Dot plot of representative marker genes across myeloid clusters. Dot size indicates the fraction of expressing cells; color reflects relative expression.

C. Box plots of CIBERSORT estimated fractions of 22 immune cell types in $Auto\text{-}RS^{high}$ (yellow) and $Auto\text{-}RS^{low}$ (green) tumors. Boxes show the interquartile range with the median; whiskers extend to 1.5× IQR. Group comparisons are by two-sided Mann-Whitney U test (*$P < 0.05$, **$P < 0.01$, ***$P < 0.001$; ns, not significant).

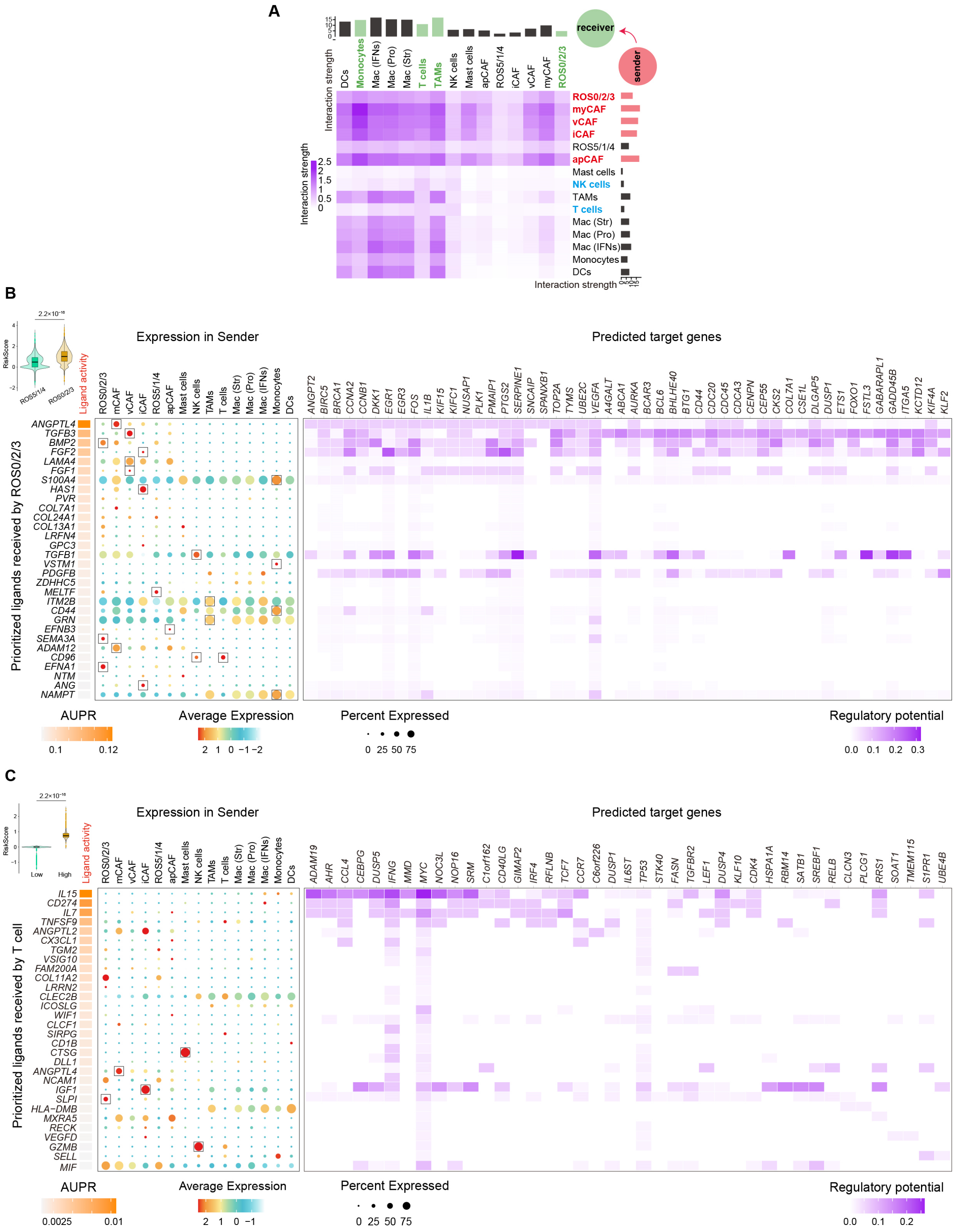
A
receiver
sender
Interaction strength
DCs
Monocytes
Mac (IFNs)
Mac (Pro)
Mac (Str)
T cells
TAMs
NK cells
Mast cells
apCAF
ROS5/1/4
iCAF
vCAF
myCAF
ROS0/2/3
2.5
2
1.5
1
0.5
0
B
Expression in Sender
Predicted target genes
Prioritized ligands received by ROS0/2/3
Ligand activity
RiskScore
ROS5/1/4
ROS0/2/3
2.2×10⁻¹⁶
ANGPTL4
TGFB3
BMP2
FGF2
LAMA4
FGF1
S100A4
HAS1
PVR
COL7A1
COL24A1
COL13A1
LRFN4
GPC3
TGFB1
VSTM1
PDGFB
ZDHHC5
MELTF
ITM2B
CD44
GRN
EFNB3
SEMA3A
ADAM12
CD96
EFNA1
NTM
ANG
NAMPT
ANGPT2
BIRC5
BRCA1
CCNA2
CCNB1
DKK1
EGR1
EGR3
FOS
IL1B
KIF15
KIFC1
NUSAP1
PLK1
PMAIP1
PTGS2
SERPINE1
SNCAIP
SPANXB1
TOP2A
TYMS
UBE2C
VEGFA
A4GALT
ABCA1
AURKA
BCAR3
BCL6
BHLHE40
BTG1
CD44
CDC20
CDC45
CDCA3
CENPN
CEP55
CKS2
COL7A1
CSE1L
DLGAP5
DUSP1
ETS1
FOXO1
FSTL3
GABARAPL1
GADD45B
ITGA5
KCTD12
KIF4A
KLF2
AUPR
0.1
0.12
Average Expression
2 1 0 −1 −2
Percent Expressed
0 25 50 75
Regulatory potential
0.0 0.1 0.2 0.3
C
Expression in Sender
Predicted target genes
Prioritized ligands received by T cell
Ligand activity
RiskScore
Low
High
2.2×10⁻¹⁶
IL15
CD274
IL7
TNFSF9
ANGPTL2
CX3CL1
TGM2
VSIG10
FAM200A
COL11A2
LRRN2
CLEC2B
ICOSLG
WIF1
CLCF1
SIRPG
CD1B
CTSG
DLL1
ANGPTL4
NCAM1
IGF1
SLPI
HLA−DMB
MXRA5
RECK
VEGFD
GZMB
SELL
MIF
ADAM19
AHR
CCL4
CEBPG
DUSP5
IFNG
MMD
MYC
NOC3L
NOP16
SRM
C1orf162
CD40LG
GIMAP2
IRF4
RFLNB
TCF7
CCR7
C6orf226
DUSP1
IL6ST
TP53
STK40
FASN
TGFBR2
LEF1
DUSP4
KLF10
CDK4
HSPA1A
RBM14
SATB1
SREBF1
RELB
CLCN3
PLCG1
RRS1
SOAT1
TMEM115
S1PR1
UBE4B
AUPR
0.0025
0.01
Average Expression
2 1 0 −1
Percent Expressed
0 25 50 75
Regulatory potential
0.0 0.1 0.2

Figure S7: Cell–cell communication analysis in the osteosarcoma microenvironment.

A, Ligand–receptor interaction strength among osteoblastic OS, CAF, myeloid, NK, and T cell subtypes. Rows represent sender cells (ordered by *Auto-RS*, low to high, bottom to top); columns represent receiver cells (ordered by *Auto-RS*, low to high, left to right).

B, NicheNet analysis of ligand–target signaling with ROS0/2/3 as receiver and surrounding clusters as senders. Left: ligands; right: predicted target genes. Black boxes highlight the specific ligand–receptor pairs discussed in the main text. The violin plot (upper left) shows *Auto-RS* distributions between ROS0/2/3 and ROS5/1/4 clusters, with ROS0/2/3 having significantly higher scores ($P < 0.001$).

C, NicheNet analysis of ligand–target signaling with T cells as receiver and surrounding clusters as senders. Left: ligands; right: predicted target genes. Black boxes highlight the specific ligand–receptor pairs discussed in the main text. The violin plot (upper left) shows *Auto-RS* distributions between low- and high-risk groups, with high-risk groups having significantly higher scores ($P < 0.001$).

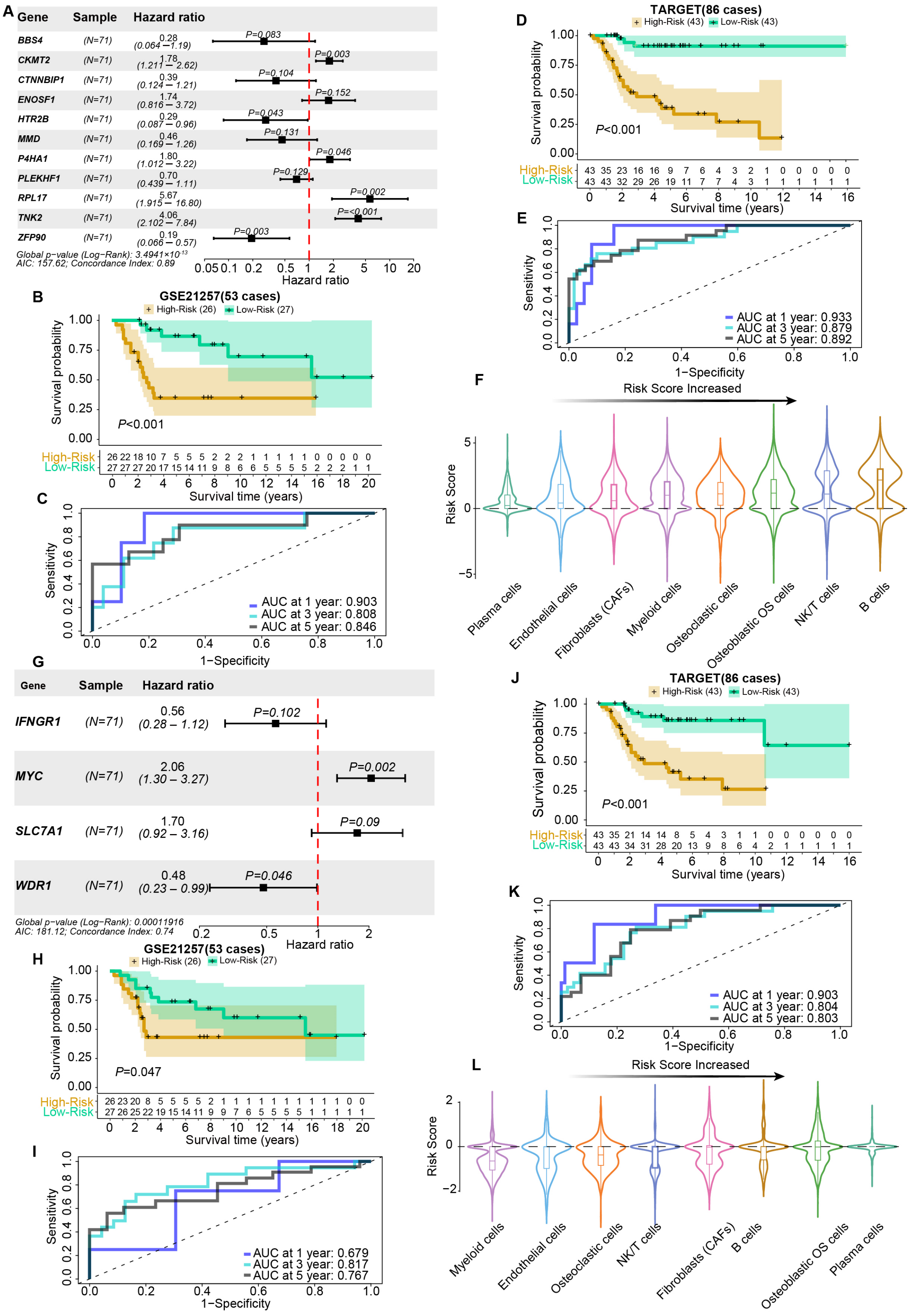

A
Gene Sample Hazard ratio
BBS4 (N=71) 0.28 (0.064 − 1.19) P=0.083
CKMT2 (N=71) 1.78 (1.211 − 2.62) P=0.003
CTNNBIP1 (N=71) 0.39 (0.124 − 1.21) P=0.104
ENOSF1 (N=71) 1.74 (0.816 − 3.72) P=0.152
HTR2B (N=71) 0.29 (0.087 − 0.96) P=0.043
MMD (N=71) 0.46 (0.169 − 1.26) P=0.131
P4HA1 (N=71) 1.80 (1.012 − 3.22) P=0.046
PLEKHF1 (N=71) 0.70 (0.439 − 1.11) P=0.129
RPL17 (N=71) 5.67 (1.915 − 16.80) P=0.002
TNK2 (N=71) 4.06 (2.102 − 7.84) P=<0.001
ZFP90 (N=71) 0.19 (0.066 − 0.57) P=0.003
Global p−value (Log−Rank): 3.4941×10⁻¹³
AIC: 157.62; Concordance Index: 0.89
Hazard ratio
B
GSE21257(53 cases)
High-Risk (26) Low-Risk (27)
Survival probability
P<0.001
High-Risk 26 22 18 10 7 5 5 5 2 2 2 1 1 1 1 1 0 0 0 0 0
Low-Risk 27 27 27 20 17 15 14 11 9 8 6 6 5 5 5 5 2 2 2 1 1
Survival time (years)
C
Sensitivity
AUC at 1 year: 0.903
AUC at 3 year: 0.808
AUC at 5 year: 0.846
1−Specificity
D
TARGET(86 cases)
High-Risk (43) Low-Risk (43)
Survival probability
P<0.001
High-Risk 43 35 23 16 16 9 7 6 4 3 2 1 0 0 0 0 0
Low-Risk 43 43 32 29 26 19 11 7 7 4 3 1 1 1 1 1 1
Survival time (years)
E
Sensitivity
AUC at 1 year: 0.933
AUC at 3 year: 0.879
AUC at 5 year: 0.892
1−Specificity
F
Risk Score Increased
Risk Score
Plasma cells
Endothelial cells
Fibroblasts (CAFs)
Myeloid cells
Osteoclastic cells
Osteoblastic OS cells
NK/T cells
B cells
G
Gene Sample Hazard ratio
IFNGR1 (N=71) 0.56 (0.28 – 1.12) P=0.102
MYC (N=71) 2.06 (1.30 – 3.27) P=0.002
SLC7A1 (N=71) 1.70 (0.92 – 3.16) P=0.09
WDR1 (N=71) 0.48 (0.23 – 0.99) P=0.046
Global p−value (Log−Rank): 0.00011916
AIC: 181.12; Concordance Index: 0.74
Hazard ratio
H
GSE21257(53 cases)
High-Risk (26) Low-Risk (27)
Survival probability
P=0.047
High-Risk 26 23 20 8 5 5 5 5 2 1 1 1 1 1 1 1 1 1 1 0 0
Low-Risk 27 26 25 22 19 15 14 11 9 9 7 6 5 5 5 5 1 1 1 1 1
Survival time (years)
I
Sensitivity
AUC at 1 year: 0.679
AUC at 3 year: 0.817
AUC at 5 year: 0.767
1−Specificity
J
TARGET(86 cases)
High-Risk (43) Low-Risk (43)
Survival probability
P<0.001
High-Risk 43 35 21 14 14 8 5 4 3 1 1 0 0 0 0 0 0
Low-Risk 43 43 34 31 28 20 13 9 8 6 4 2 1 1 1 1 1
Survival time (years)
K
Sensitivity
AUC at 1 year: 0.903
AUC at 3 year: 0.804
AUC at 5 year: 0.803
1−Specificity
L
Risk Score Increased
Risk Score
Myeloid cells
Endothelial cells
Osteoclastic cells
NK/T cells
Fibroblasts (CAFs)
B cells
Osteoblastic OS cells
Plasma cells

Figure S8: Model analysis of genes enriched in the whole transcriptome and tumor-related metastasis pathways.

A, Forest plot of 11 whole-transcriptome genes identified by multivariate Cox regression. Horizontal lines show 95% confidence intervals (CI) of hazard ratios. The red dashed line marks hazard ratios = 1 (no effect), > 1 indicates risk, < 1 indicates protection. *P* values were derived from the Wald test.

B,**C**, Kaplan–Meier survival curves (**B**) and ROC analysis (**C**) of the predictive model in GSE21257 (*n* = 53). High-risk patients showed significantly shorter survival (log-rank test), with ROC curves indicating good predictive accuracy at 1, 3, and 5 years.

D,**E**, Kaplan–Meier survival curves (**D**) and ROC analysis (**E**) in TARGET (*n* = 86), showing consistent results.

F, *Auto-RS* based on the 11-gene model applied to single-cell data. Distribution of *Auto-RS* across eight major cell populations, increasing from left to right.

G, Forest plot of four metastasis-related genes identified by multivariate Cox regression Horizontal lines show 95% confidence intervals (CI) of hazard ratios. The red dashed line marks hazard ratios = 1 (no effect), > 1 indicates risk, < 1 indicates protection. *P* values were derived from the Wald test.

H,**I**, Kaplan–Meier survival curves (**H**) and ROC analysis (**I**) of the 4-gene model in GSE21257 (*n* = 53). High-risk patients showed significantly shorter survival (log-rank test), with ROC curves indicating good predictive accuracy at 1, 3, and 5 years.

J,**K**, Kaplan–Meier survival curves (**J**) and ROC analysis (**K**) in TARGET (*n* = 86). High-risk patients showed significantly shorter survival (log-rank test), with ROC curves indicating good predictive accuracy at 1, 3, and 5 years.

L, *Auto-RS* based on the 4-gene model applied to single-cell data. *Auto-RS* distribution across eight major cell populations, increasing from left to right.

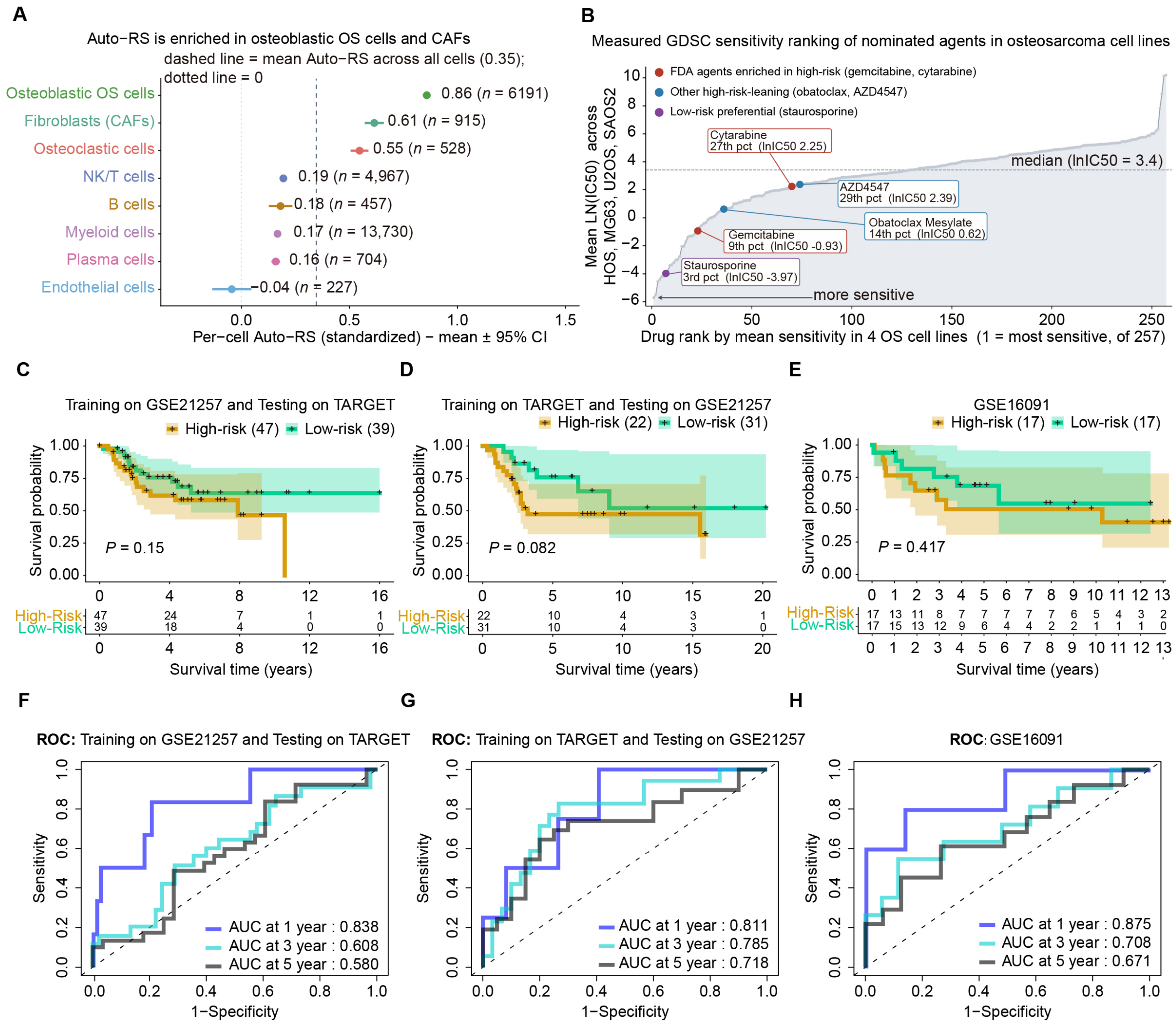

Figure S9: Per cell type *Auto-RS* enrichment, GDSC sensitivity ranking of nominated agents, and strict external validation of the Auto-RS signature.

A, Per-cell *Auto-RS* across the integrated single-cell dataset, computed from the four-gene Cox-weighted formula and standardized. Points show the mean ± 95% CI for each cell type; the dashed line marks the mean *Auto-RS* across all cells (0.35) and the dotted line marks zero. Osteoblastic OS cells (0.86, *n* = 6191) and cancer-associated fibroblasts (0.61, *n* = 915) carried the highest scores and were significantly elevated relative to all other populations pooled (one-sided Wilcoxon rank-sum, BH-adjusted $P < 2.2 \times 10^{-16}$; Kruskal-Wallis $P < 2.2 \times 10^{-16}$ across cell types).

B, Ranking of the nominated agents within the experimentally measured GDSC sensitivity profile of the four OS cell lines (HOS, MG63, U2OS, SAOS2). Drugs are ordered by mean

sensitivity across the four lines (rank 1 = most sensitive, of 257 profiled compounds); the y-axis shows the mean ln(IC50) and a lower value indicates greater sensitivity. The dashed line marks the median ln(IC50) (3.4). Gemcitabine (9th percentile, ln IC50 −0.93) and cytarabine (27th percentile, ln IC50 2.25) rank among the more potent agents, alongside obatoclax (14th percentile) and AZD4547 (29th percentile), whereas staurosporine (3rd percentile) is the low-risk-preferential nomination.

C–E, Kaplan-Meier overall-survival curves for median-dichotomized high- and low-risk groups under strict lock-and-transfer external validation: (C) training on GSE21257 and testing on TARGET (high-risk n = 47, low-risk n = 39; log-rank P = 0.15); (D) training on TARGET and testing on GSE21257 (high-risk n = 22, low-risk n = 31; log-rank P = 0.082); and (E) the fully independent GSE16091 cohort (high-risk n = 17, low-risk n = 17; log-rank P = 0.417). Shaded bands denote 95% CIs; risk tables are shown below each curve.

,F–H, Time-dependent ROC curves for the continuous *Auto-RS* at 1, 3, and 5 years, corresponding to the three validations above: (F) GSE21257 to TARGET (AUC 0.84, 0.60, 0.58); (G) TARGET to GSE21257 (AUC 0.81, 0.78, 0.72); and (H) GSE16091 (AUC 0.875, 0.708, 0.671).
