# Supplementary figures and images for: An Auto-RS Signature for Prognostic Stratification and Drug Sensitivity Prediction in Osteosarcoma
Source: Genes (Basel). 2026 Jun 26;17(7):737. doi: 10.3390/genes17070737 (PMC13408425; doi:10.3390/genes17070737)

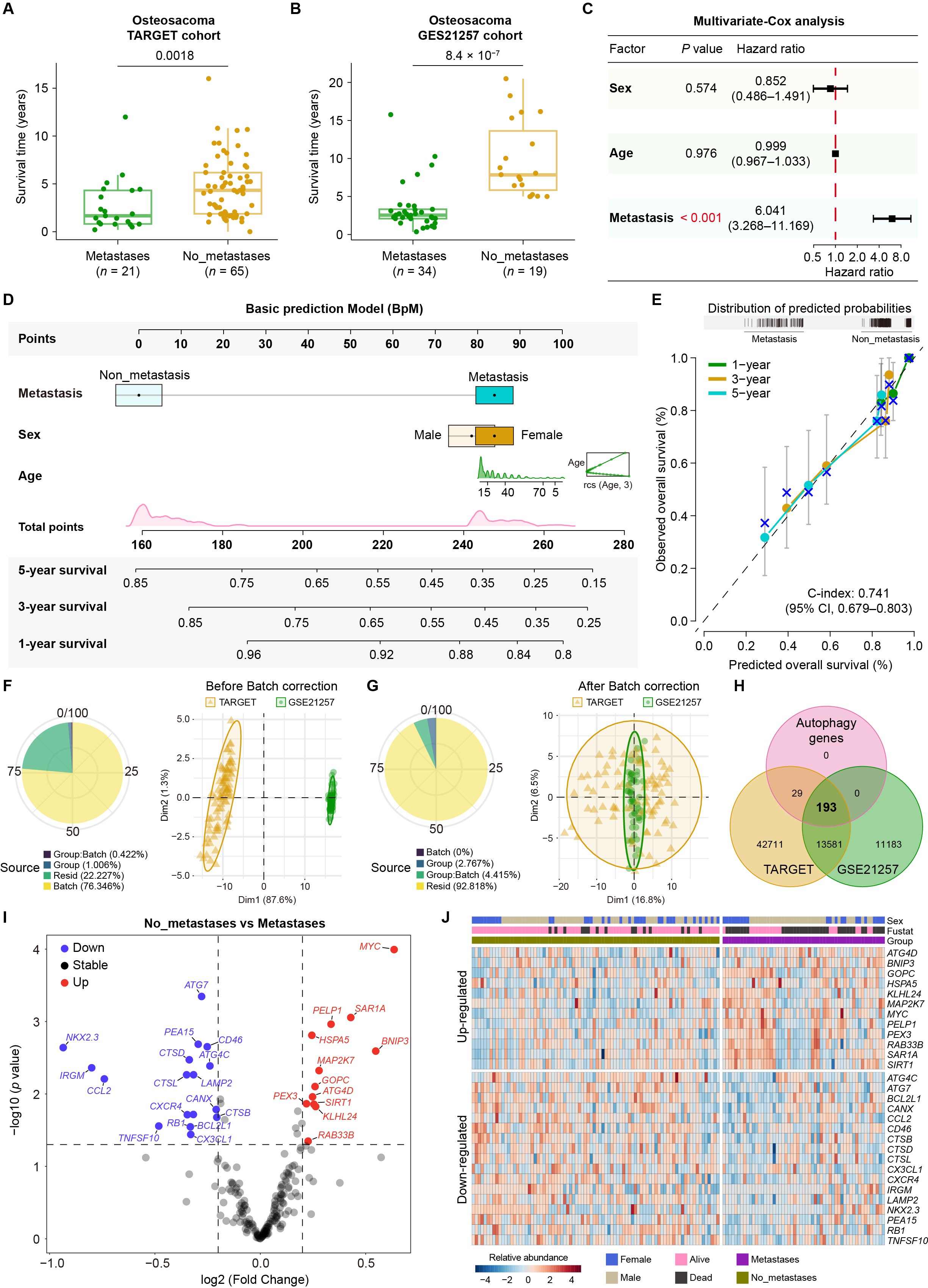

Supplement: Supplementary file 1 [file genes-17-00737-s001.zip › Figure S1.jpg]

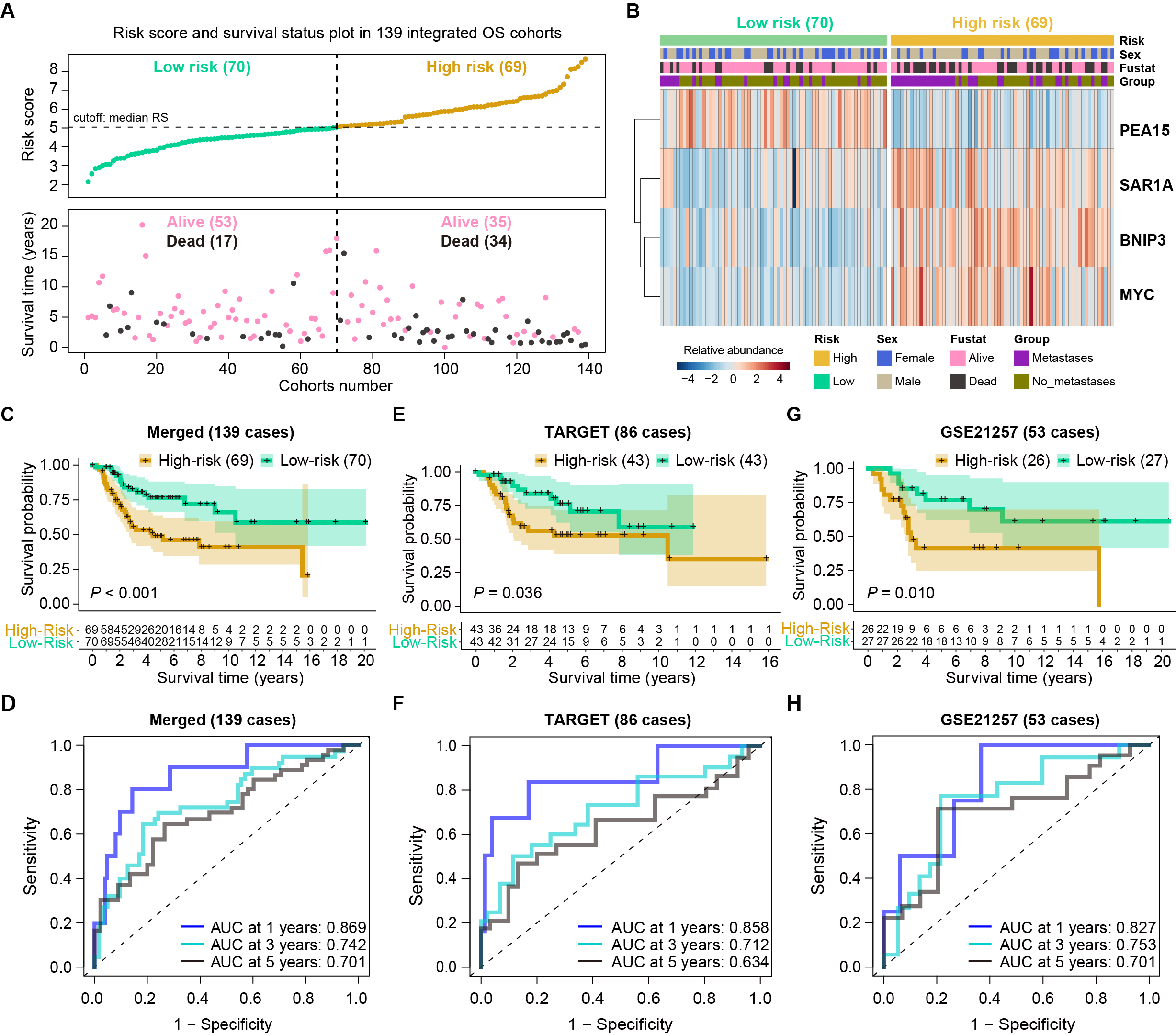

Supplement: Supplementary file 1 [file genes-17-00737-s001.zip › Figure S2.jpg]

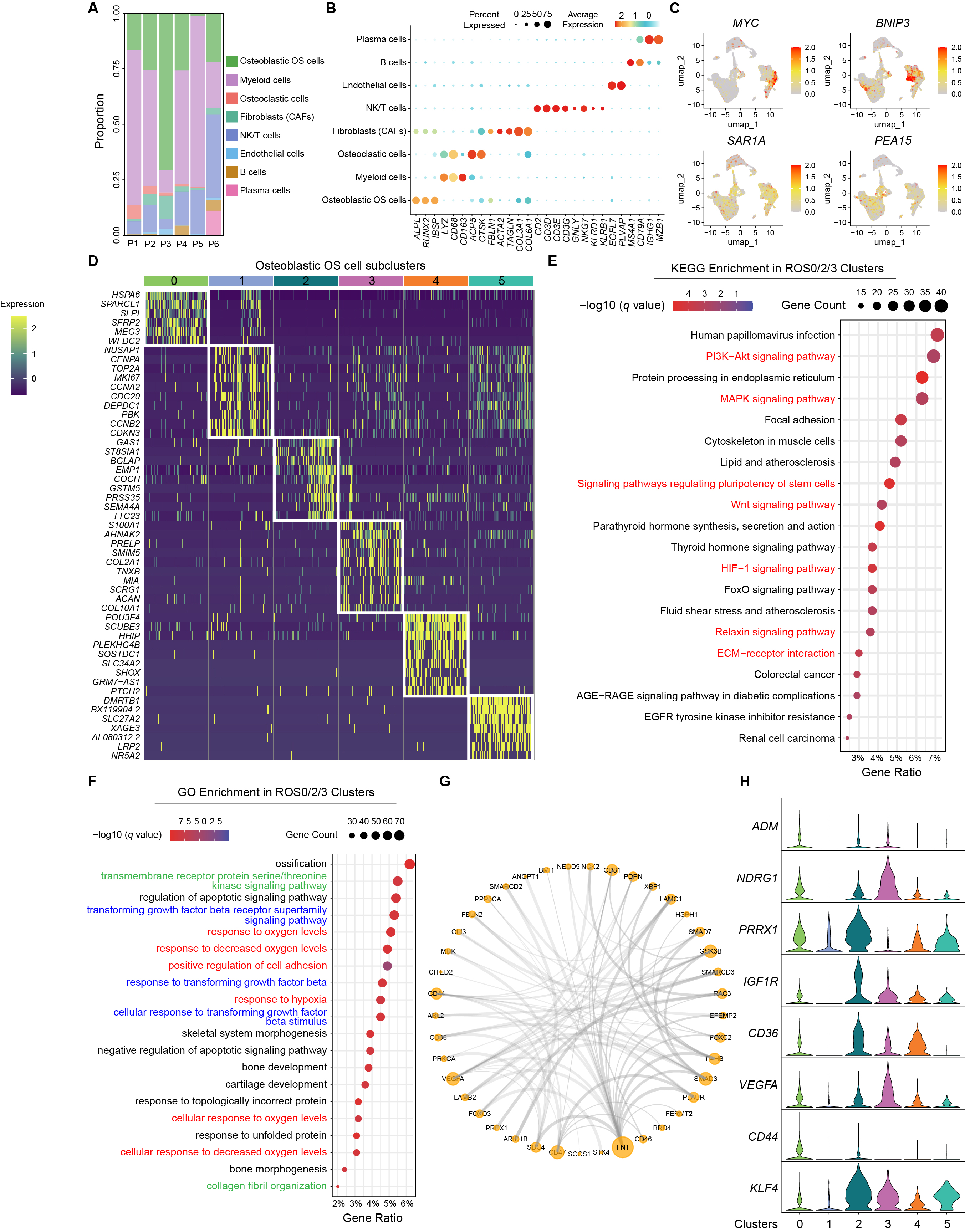

Supplement: Supplementary file 1 [file genes-17-00737-s001.zip › Figure S3.jpg]

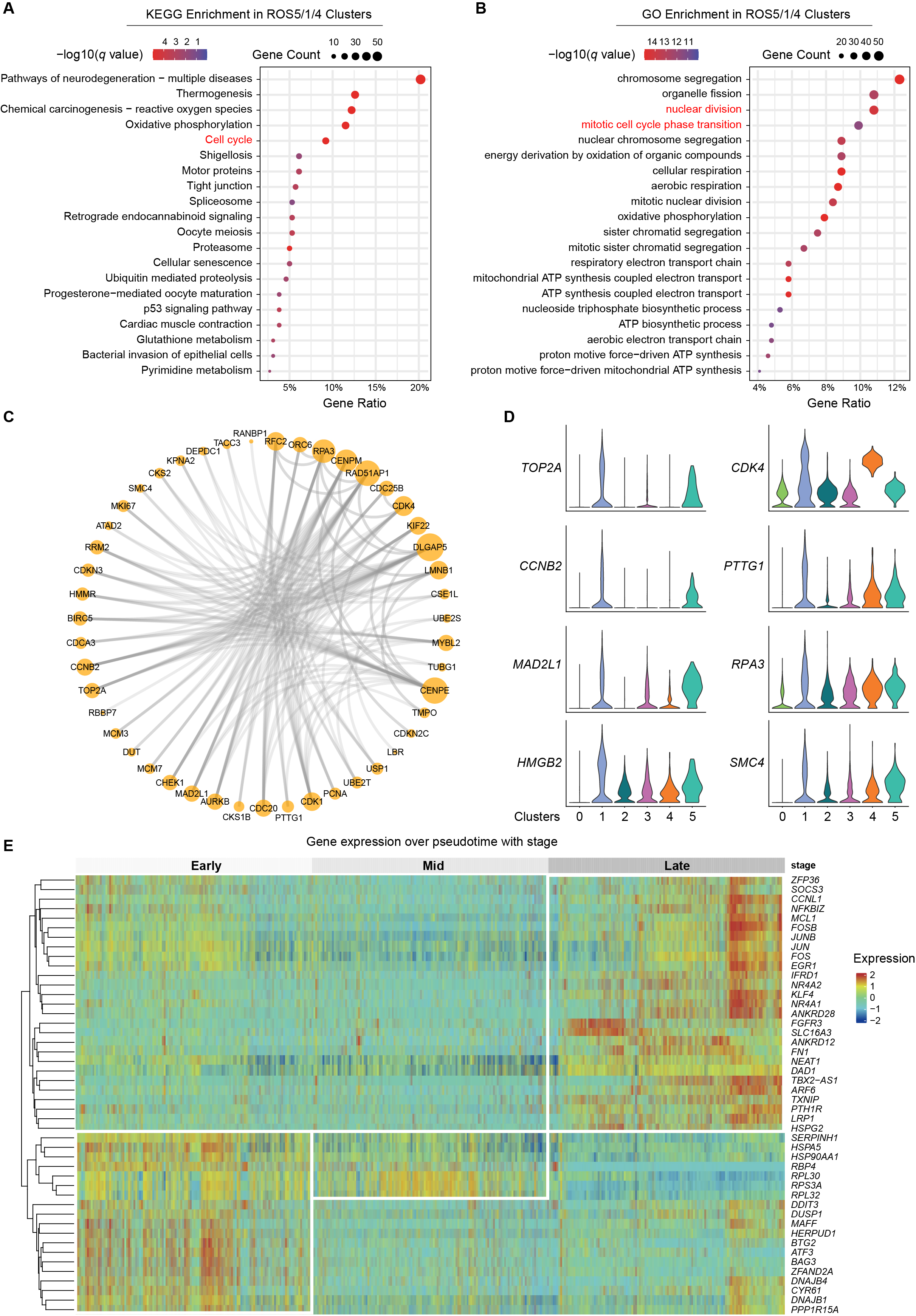

Supplement: Supplementary file 1 [file genes-17-00737-s001.zip › Figure S4.jpg]

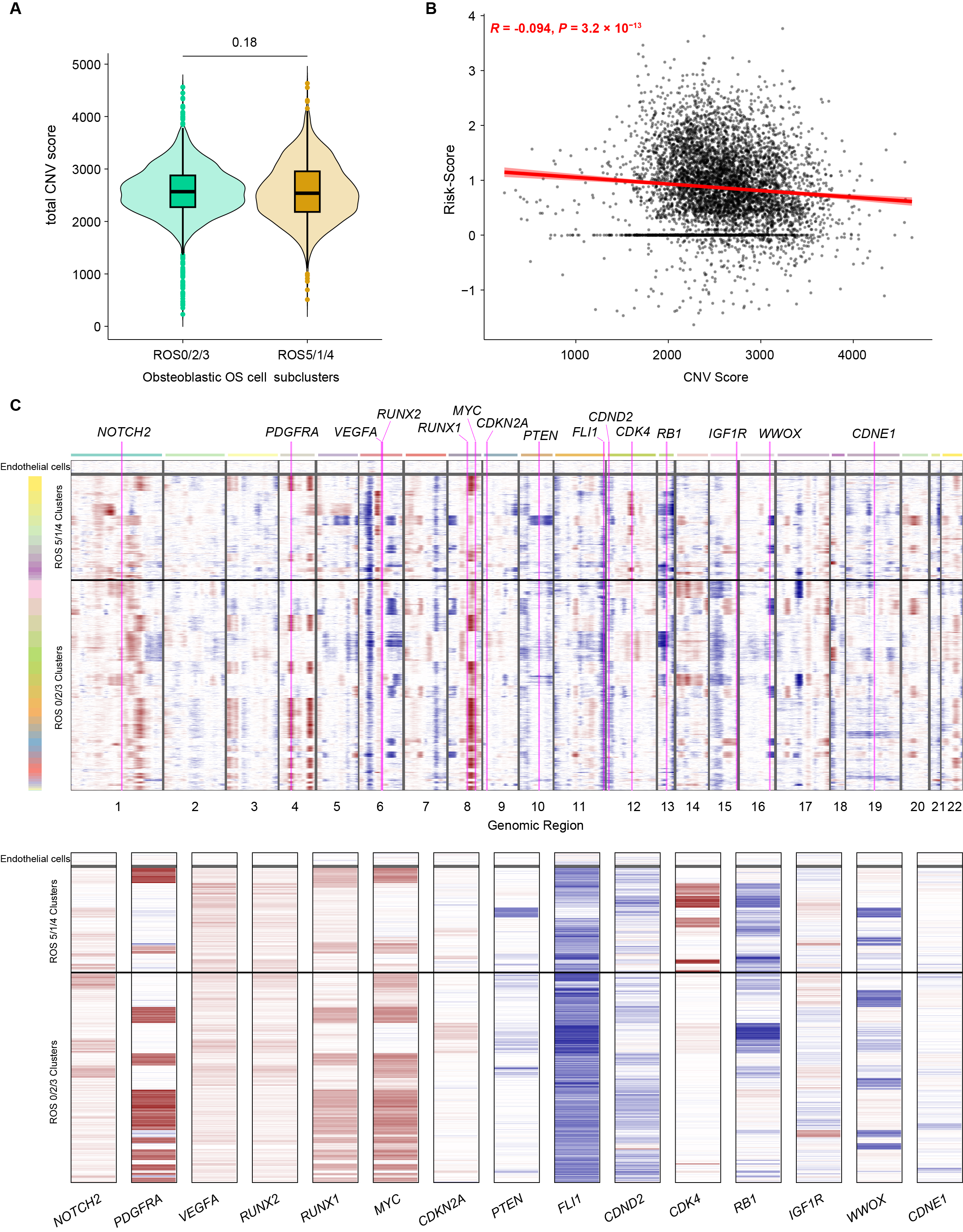

Supplement: Supplementary file 1 [file genes-17-00737-s001.zip › Figure S5.jpg]

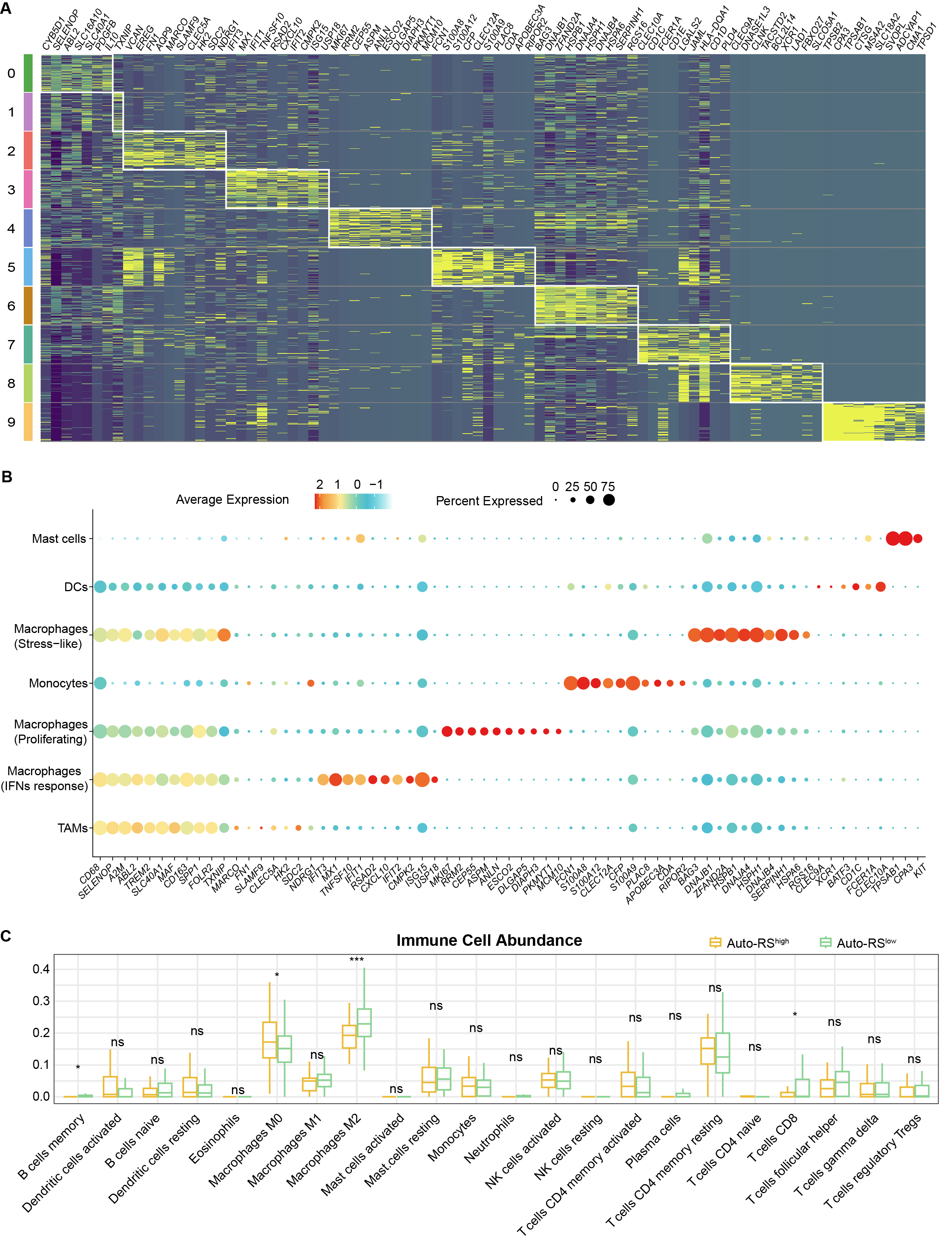

Supplement: Supplementary file 1 [file genes-17-00737-s001.zip › Figure S6.jpg]

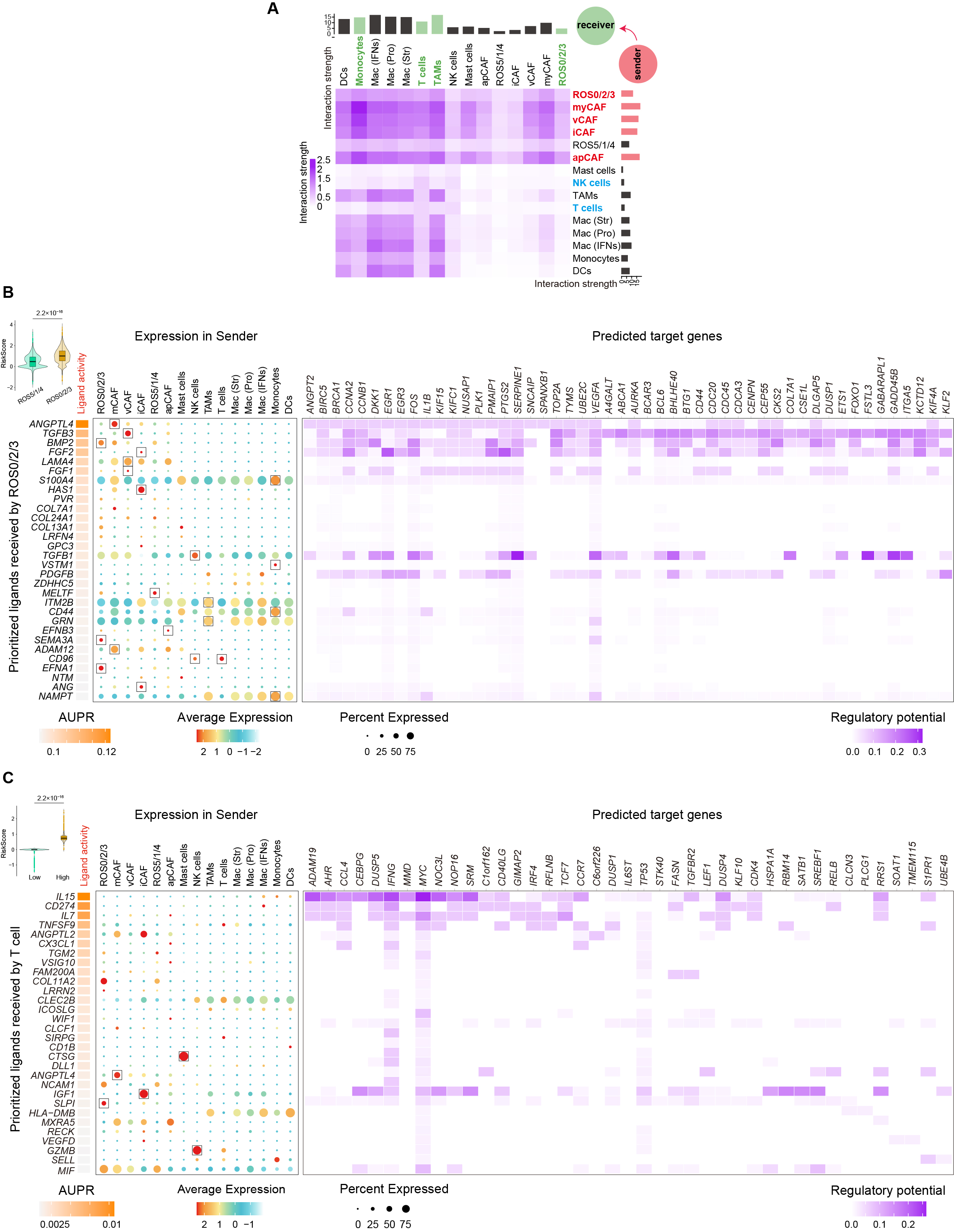

Supplement: Supplementary file 1 [file genes-17-00737-s001.zip › Figure S7.jpg]

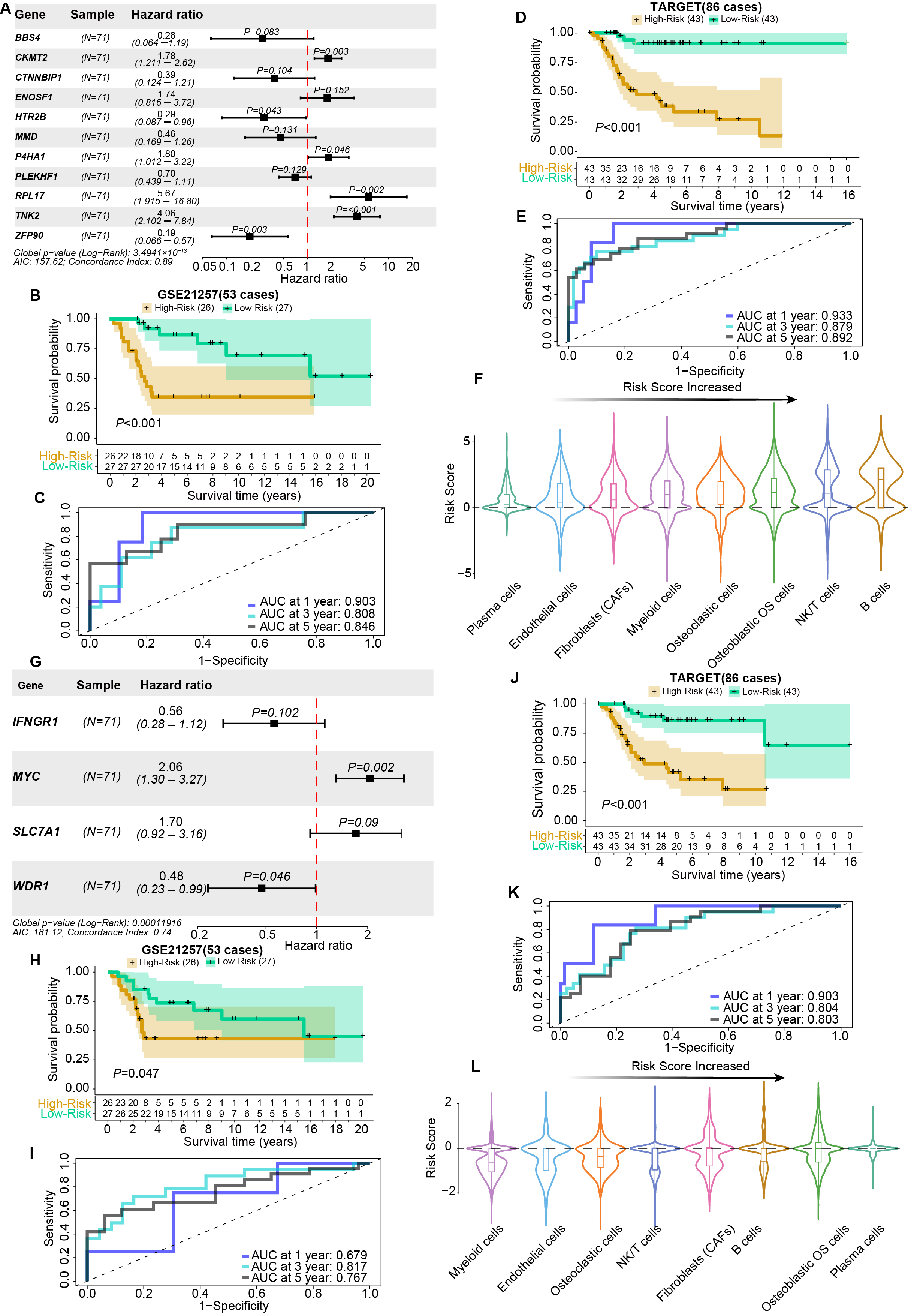

Supplement: Supplementary file 1 [file genes-17-00737-s001.zip › Figure S8.jpg]

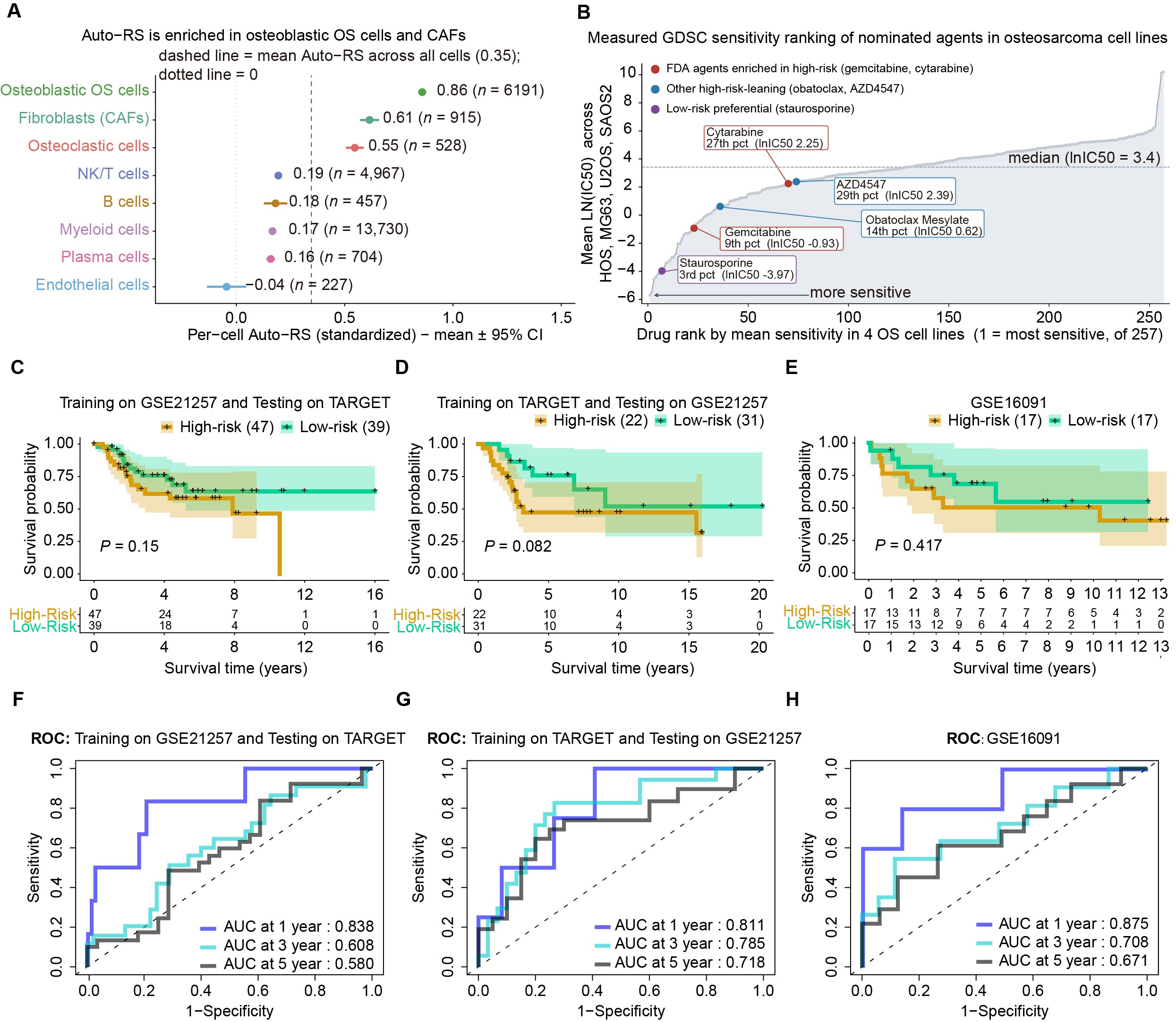

Supplement: Supplementary file 1 [file genes-17-00737-s001.zip › Figure S9.jpg]
